# Stereo slant discrimination of planar 3D surfaces: Frontoparallel versus planar matching

**DOI:** 10.1167/jov.22.5.6

**Published:** 2022-04-25

**Authors:** Can Oluk, Kathryn Bonnen, Johannes Burge, Lawrence K. Cormack, Wilson S. Geisler

**Affiliations:** 1 Center for Perceptual Systems and Department of Psychology, University of Texas at Austin, Austin, TX, USA; 2 School of Optometry, Indiana University Bloomington, Bloomington, IN, USA; 3 Department of Psychology, University of Pennsylvania, Philadelphia, PA, USA

**Keywords:** stereo slant discrimination, 3D surface orientation, ideal observer, cross correlation

## Abstract

Binocular stereo cues are important for discriminating 3D surface orientation, especially at near distances. We devised a single-interval task where observers discriminated the slant of a densely textured planar test surface relative to a textured planar surround reference surface. Although surfaces were rendered with correct perspective, the stimuli were designed so that the binocular cues dominated performance. Slant discrimination performance was measured as a function of the reference slant and the level of uncorrelated white noise added to the test-plane images in the left and right eyes. We compared human performance with an approximate ideal observer (planar matching [PM]) and two subideal observers. The PM observer uses the image in one eye and back projection to predict a test image in the other eye for all possible slants, tilts, and distances. The estimated slant, tilt, and distance are determined by the prediction that most closely matches the measured image in the other eye. The first subideal observer (local planar matching [LPM]) applies PM over local neighborhoods and then pools estimates across the test plane. The second suboptimal observer (local frontoparallel matching [LFM]) uses only location disparity. We find that the ideal observer (PM) and the first subideal observer (LPM) outperforms the second subideal observer (LFM), demonstrating the additional benefit of pattern disparities. We also find that all three model observers can account for human performance, if two free parameters are included: a fixed small level of internal estimation noise, and a fixed overall efficiency scalar on slant discriminability.

## Introduction

**Figure 1.**
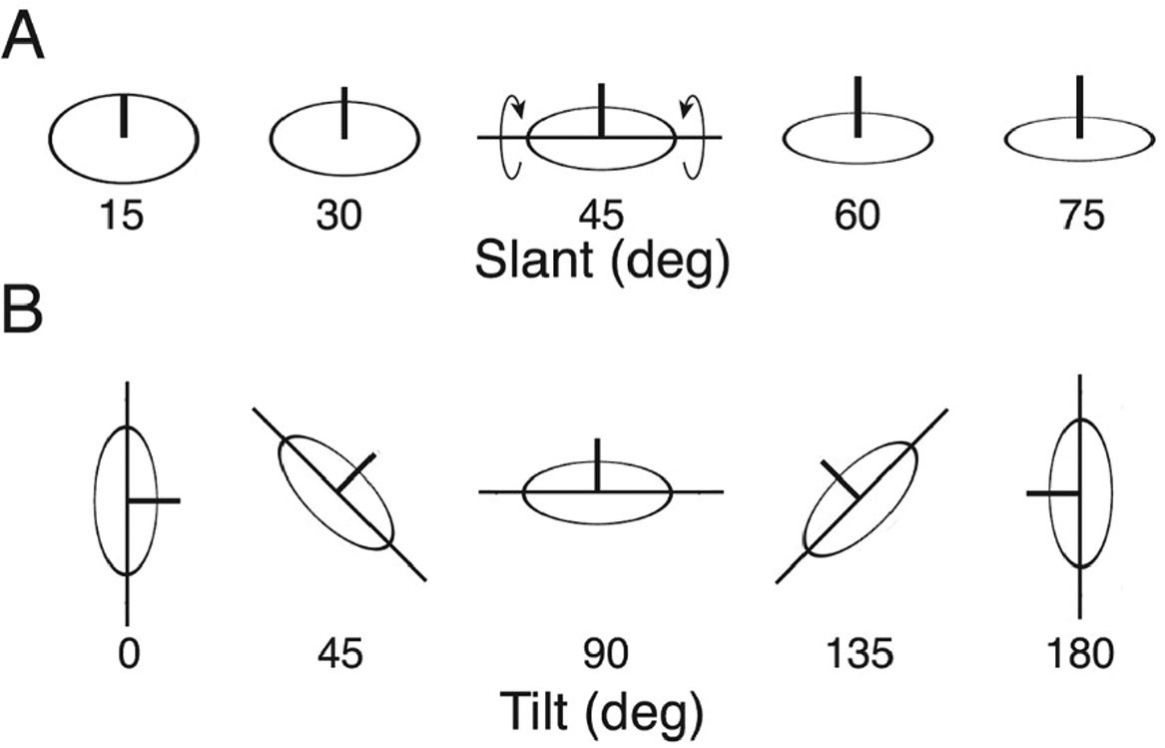
Definition of slant and tilt. (**A**) Slant is the angle between the surface normal (black vertical line segment) and the frontoparallel plane. Here, the slant is varied while the tilt remains at 90 degrees. (**B**) Tilt is the orientation of the vector formed by the projection of the surface normal onto the frontoparallel plane. Here, the tilt is varied while the slant remains at 45 degrees.

Estimating the 3D shape of our surroundings is essential for many everyday behaviors. The 3D shape at any point on a smooth surface can be closely approximated over a small neighborhood by a plane. Thus, the most local and fundamental measure of shape is local surface orientation. Local surface orientation is often specified in terms of slant and tilt (Stevens, 1983). Slant is the angle between the surface normal (the unit vector perpendicular to the surface) and the frontoparallel plane (Figure 1A). Tilt is the orientation of the vector formed by the projection of the surface normal onto the frontoparallel plane (Figure 1B).

A common view of 3D shape perception is that it begins with the estimation of the local slants and tilts, which are then integrated into a representation of the 3D shape. Thus, not surprisingly, there have been a large number of studies directed at measuring and understanding the perception of 3D slant and tilt (e.g. see Howard & Rogers, 2012). Here, we focus on perception of the 3D slant of planar surfaces.

Under natural conditions (without head or scene movement), the image information available for 3D slant estimation typically consists of the binocular cue of disparity (the differences between the images formed in the two eyes) together with various monocular cues (e.g. linear perspective). The primary goal of the current study was to measure slant discrimination under naturalistic conditions and to compare human performance in our task with that of an ideal observer, and several subideal observers, for slant estimation from binocular-disparity cues.

**Figure 2.**
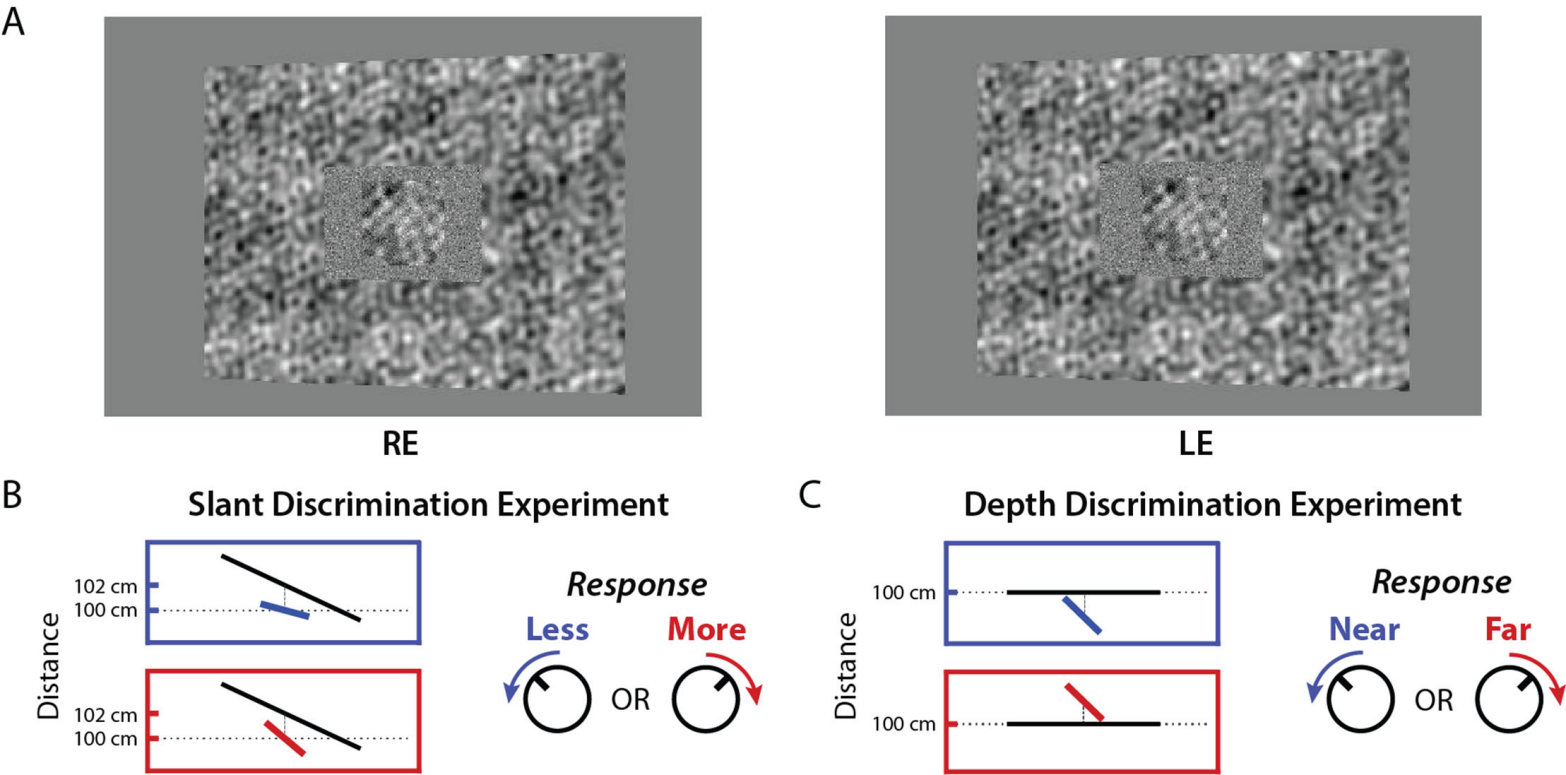
Stimuli and task in the slant and depth discrimination experiments. (**A**) Example binocular stimulus (crossed). The actual stimuli were presented in a stereo rig where orthogonally polarized left- and right-eye images alternated at 60 Hz, and were viewed through polarization-selective filters. The rectangular reference plane was densely textured, had a central window/hole, and was rendered at a distance of 102 cm. The central test plane was a trapezoid jittered in distance and aspect ratio to reduce the usability of monocular perspective cues. On each trial, an independent sample of white noise was added to the window region in the left- and right-eye images. (**B**) As illustrated in a top-down view, the subjects’ task was to judge whether the central test plane was more or less slanted than the reference plane. Subjects had unlimited viewing time and responded by rotating a knob clockwise or counterclockwise. (**C**) Top down view of the depth discrimination experiment. The reference plane was frontoparallel and the subject judged whether the slanted test plane was near or far.

A number of studies have measured slant-discrimination performance from binocular disparity using sparse random dot stereograms (Hibbard, Bradshaw, Langley, & Rogers, 2002; Knill & Saunders, 2003; Hillis, Watt, Landy, & Banks, 2004; Girshick & Banks, 2009; Burge, Girshick, & Banks, 2010). In most of these studies, the stimuli were presented in two temporal intervals. In natural viewing, it is probably more typical for humans to be comparing the 3D orientations of surfaces that are densely textured, and that are located within the same scene at different distances (Burge, McCann, & Geisler, 2016; Kim & Burge, 2018; Kim & Burge, 2020). Here, we measured slant discrimination performance for surfaces that were textured with naturalistic noise (see Figure 2 in Methods). The two planar surfaces were presented in a single-interval task, where the smaller test surface was in front of a surrounding reference surface by a distance that varied randomly from trial-to-trial by a small amount. The stimuli were accurately rendered, and hence contained both monocular and binocular cues to surface orientation. To reduce the usefulness of the monocular cues, the texture contained few regularities and the shape (i.e. silhouette) of the test surface was jittered (see Methods). A control experiment confirmed that the performance of our subjects was completely dominated by the binocular cues (see Results). This allowed us to focus on models of slant discrimination from binocular disparity. Finally, to limit and compare human and model-observer performance, we added different (uncorrelated) samples of white noise to the test region in each eye.

The modeling begins with the derivation of an approximate Bayesian ideal observer for slant discrimination of planar surfaces from binocular disparity. Ideal-observer models reveal the fundamental computational principles of the task, set a proper benchmark to compare with human performance, and can be used to evaluate the effectiveness of heuristic (suboptimal) mechanisms (Green & Swets, 1966; Geisler, 2011; Burge, 2020).

In the Bayesian framework, it is convenient to divide the problem of estimating slant from binocular disparity into two problems (e.g. Marr & Poggio, 1979). The first is the “correspondence problem”: estimating the points in the left image and the points in the right image that correspond to the same points in the 3D scene. Here, we define the transformation that maps one image into the other as the “disparity” between the two images. It is important to note that there are multiple ways to describe the same transformation. For example, a disparity might be most compactly described as a global transformation with just a few parameters, but can also be described by a list of the vertical and horizontal translations of each point in one image needed to align that point with the corresponding point in the other image. Solving the correspondence problem can be difficult because of false matches and partial occlusions.

The second problem is to translate the estimated disparity, or disparities, into an estimate of the 3D surface orientation. Solving this problem requires knowing or estimating the pose of the eyes (e.g. vergence and version), which may be estimated from image cues, oculomotor cues, head orientation cues, or some combination of these cues.

The ideal and subideal observers described here assume that the pose of the eyes is known and hence the current focus is more on the correspondence problem. This focus differs from related human vision literature, which assumes that the correspondence problem has been solved and focuses instead on the estimation of surface orientation from cues in the matched binocular images (such as the horizontal and vertical size ratio, and disparity gradients), and from other eye-pose cues (e.g. see Backus, Banks, van Ee, & Crowell, 1999).

For every possible slant and distance of the surface, the ideal observer computes the predicted image in one eye given the image observed in the other eye and the rules of backward- and forward-projection (see Methods). The estimated slant and distance are the slant and distance pair that gives the smallest prediction error. We will call this optimal model of slant and distance estimation the “planar matching” (PM) model. This observer is optimal because it uses all of the available geometric information given planar surfaces. By generating predictions via backward and forward projection for every possible distance and slant, the PM observer is considering exactly the set of possible differences that can exist between the left and right images for a given planar surface. It then picks the distance and slant that best explains the difference between the two images, and thus it simultaneously solves the correspondence and slant-estimation problems with the estimation of just two global parameters.

Although, for simplicity, we assume that eye pose is known and that the model observers compute absolute slant and distance, we argue later that there are nearly equivalent model observers that compute relative slant and are robust to modest uncertainty in eye pose (see Methods and Discussion).

In general, human performance deviates from optimal performance. A principled approach for generating plausible suboptimal models is to replace one or more of the optimal computations with simpler more biologically plausible computations, to incorporate sources of internal noise, and/or to incorporate other plausible biological limitations (e.g. response nonlinearities, foveation, etc.).

One simplifying and more biologically plausible computation is to perform PM locally to solve the local correspondence problem, and then combine the local slant and distance estimates over the whole test region (Jones & Malik, 1992; Super & Klarquist, 1997; see also Wildes, 1991). We will call this the “local planar matching” (LPM) model. The LPM model represents the correspondence between local image regions in the two eyes as a “structural disparity”—a difference in the location and spatial pattern between the corresponding regions in the two eyes (location disparities and pattern disparities). To be clear, in this paper, we regard structural disparities as the result of an initial binocular matching process, and not as second-level cues (like horizontal and vertical size ratios) computed after the correspondence problem has been solved.

A more simplifying assumption is to solve the local correspondence problem by performing “local frontoparallel matching” (LFM), which is essentially equivalent to standard cross correlation (Tyler & Julesz, 1978; Cormack, Stevenson, & Schor, 1991; Banks, Gepshtein, & Landy, 2004). The LFM model represents the correspondence between local image regions in the two eyes as only a “location disparity”—the difference in the location of the corresponding regions in the two eyes. This assumption is made by most models of human stereo vision. The estimated surface slant is then computed by combining the distances specified by the location disparities. Formally, this LFM model is a special case of the LPM model where the local slant is assumed to be zero (see Methods). Models based on LFM have been successful in accounting for many aspects of human disparity discrimination (Tyler & Julesz, 1978; Cormack et al., 1991; Banks et al., 2004; Filippini & Banks, 2009), and in explaining the response properties of disparity-selective neurons in visual cortex (Ohzawa, DeAngelis, & Freeman, 1990; DeAngelis, Ohzawa & Freeman, 1991; Ohzawa, DeAngelis, & Freeman, 1997; Cumming & DeAngelis, 2001).

It has long been known that introducing an orientation or scale difference between the left and right images can produce vivid perceptions of surface slant (Wheatstone, 1838; Ogle, 1938; Blakemore, 1970a). These results seem to suggest that local structural disparities are directly exploited by the visual system to estimate 3D surface orientation. However, as mentioned above, these structural disparities can also be described in terms of the location disparities between the corresponding points in each image. Thus, it has been difficult to rule out the hypothesis that location disparities are computed first and then later combined to determine the 3D surface orientation (Fiorentini & Maffei, 1971; Wilson, 1976; Mitchison & McKee, 1990; Cagenello & Rogers, 1993; Halpern, Wilson, & Blake, 1996; Greenwald & Knill, 2009). One aim of the current study was to discriminate between these hypotheses.

In the current study, we measured slant discrimination thresholds for the human and model observers as a function of the reference slant and the contrast of uncorrelated white noise samples added to each eye's image. As expected, we found that the PM model had the lowest thresholds, followed by the LPM model, the LFM model, and finally the human subjects. All three models capture the qualitative trends in the human thresholds, but none provide good quantitative predictions of the trends, even when their average sensitivities (*d*′ values) are scaled by an arbitrary efficiency parameter. However, if we include another plausible factor, a fixed level of internal estimation noise, then all three models make good quantitative predictions. Although the LPM observer does not predict the pattern of human thresholds significantly better than the LFM observer, its absolute performance is substantially better and more robust across analysis patch size (e.g. receptive field size), and thus there may have been evolutionary pressure to incorporate similar structural-disparity computations into the early visual system. We also measured depth discrimination, in addition to slant discrimination, with the same stimuli and found that there was a trend for human observers to be more efficient (relative to ideal) at slant discrimination than at depth discrimination.

## Methods

### Subjects

Three experienced psychophysical observers (two men and one woman) served as subjects. They each had normal or corrected to normal spatial and stereo acuity. Written informed consent was obtained for all observers in accordance with The University of Texas at Austin Institutional Review Board.

### Apparatus

Stimuli were presented using a Planar PX2611W stereoscopic display (Planar Systems, Beaverton, OR, USA). This display consists of two monitors with orthogonal linear polarization relative to each other, separated by a polarization-preserving beam splitter. Subjects wore passive linearizing filters to view binocular stereo stimuli. In all experiments, subjects used a forehead rest to maintain constant viewing distance. Each monitor was gamma-corrected to produce a linear relationship between pixel values and output luminance. Luminance was measured with a photometer (PR 655; Photo Research, Syracuse, NY, USA) through the beam splitter and a polarizing lens. The background luminance of the two monitors was 46.73 and 52.04 cd/m^2^. All experiments and analyses were done using custom code written in MATLAB using the Psychophysics Toolbox (Brainard, 1997; Pelli, 1997).

### Stimuli

The stimuli (Figure 2) were stereo images centered on a display screen located at a distance of 100 cm from the eyes. The stimulus consisted of two surfaces: a relatively large rectangular surround reference plane (with an angular central height of 9 degrees), and a smaller central test plane (see Figure 2A). The slants of both the reference and test planes were varied; the tilts of the reference and test planes were always zero (i.e. both surfaces were slanted about a vertical axis). In 3D space, the rectangular reference plane had a fixed width (23 cm) and height (16 cm), and the center of the reference plane was located at a distance of 102 cm (i.e. 2 cm behind the display screen). There was a “window” (hole) in the reference plane that subtended 4.2 degrees × 3.1 degrees in the right eye, independent of the slant of the reference plane. Thus, the window size varied in the left eye when the slant of the reference plane was varied. The test plane was centered in the window of the reference plane. On each trial, an independent sample of spatial Gaussian white noise was added to the window regions of the left and right eye images. The reference slant and additive noise contrast were parametrically varied: reference slants = 0 degrees, ± 12.5 degrees, ± 25 degrees, and ± 50 degrees; root mean square (RMS) noise contrasts = 5%, 17.5%, and 34%. The slant of the test plane was varied to obtain psychometric functions for slant discrimination (see below). The stereo images were rendered assuming an interocular distance of 6.5 cm (the interocular distances of the 3 subjects are 6.5, 6.5, and 6.2 cm).

When a planar surface is slanted, monocular slant cues are created due to perspective projection. Our main interest here was in the stereo cues, and thus steps were taken to reduce the usefulness of the monocular cues. The effectiveness of these steps was confirmed with a monocular control experiment (see below). First, we randomly varied the distance of the test plane from 99 to 101 cm across trials, thereby jittering its retinal image size. Second, we fixed the width and central height of the test plane in the right eye to 2.2 degrees of visual angle and jittered the ratio of the left and right edge heights of the test plane. This jitter creates 3D test plane surfaces that are trapezoidal in shape. The average edge-height ratio was set equal to what would be expected for a rectangle having the same slant as the reference plane. The range of the random jitter around this ratio matched the range of ratios expected for a rectangular test plane varying over the range of test slants used to measure the psychometric function with the steepest slant (50 degrees). This procedure strongly reduces the usefulness of monocular shape cues to slant estimation, while introducing a relatively small amount of jitter. Even with the jitter, some monocular cues for slant remained because the surface texture in the test plane was accurately rendered.

The texture of the planes was generated by summing 59 sinewaves having spatial frequencies of constant amplitude ranging between 0.1 and 3 cycles per degree (cpd) in steps of 0.05 cpd, with the phase and orientation of each sinewave randomly sampled from a uniform distribution over all possible values (0 to π). The advantage of creating textures by summing sinewaves is that it is possible to avoid interpolation artifacts. For each pixel location, for each eye, there is an exact formula for the real-valued gray level for each sinewave component on any 3D planar surface. Given these values, we then summed the gray levels for all the components to obtain the exact real-valued gray level at each image pixel location, and then gamma-compressed and appropriately quantized them for presentation. The textures on the reference and test planes were generated separately, and both had an average RMS contrast of 14.7% before the addition of the uncorrelated white noise.

One difference between the task used here and tasks used in previous investigations of slant discrimination is that this task is arguably more typical of natural conditions. Typically, the two surfaces whose slants are being compared are presented in two different temporal intervals (Knill & Saunders, 2003; Hillis et al., 2004; Girshick & Banks, 2009). Under natural conditions, surfaces that are being compared are often at different distances and are often viewed simultaneously. In addition, in order to isolate binocular disparity cues, many previous studies have used sparse random-dot stereograms. However, most natural surfaces have a dense irregular texture (e.g. tree bark).

### Procedure

The experiment was performed under free-viewing conditions without a fixation point, although a chin and head rest were used to fix the head position. Observers were asked to indicate, in a forced choice task, whether the test plane was more or less slanted than the reference plane. To indicate their decision, observers simply turned a knob (PowerMate wireless controller; Griffin Technology, Irvine, CA, USA) in the direction in which the test plane was rotated relative to the reference plane. Observers found this method of response much more intuitive than a keypress. The stimulus was present until the observer made a response. The average trial duration was 4.7 seconds. The next stimulus appeared after a 1.5 second blank interval.

Each observer completed eight experimental sessions, where the magnitude of the reference slant was held fixed at one of the four values (± 0 degrees, ± 12.5 degrees, ± 25 degrees, and ± 50 degrees). Each session consisted of four blocks of trials. The first block was a practice block where the number of trials was half that of the other blocks and with feedback given on each trial. Practice blocks were not included in the data analysis. No feedback was given in the remaining three experimental blocks (160 trials per block). In each of these three blocks, the noise contrast was fixed at one of the three values (5%, 17.5%, or 34%). Within a session, the order of these blocks was either ascending or descending, and in the later repeat of that session the order of the noise contrasts was reversed. All 160 trials in a block had a fixed reference slant magnitude, but the sign of the reference slant was different for the first and second halves of the trials in the block (e.g. 25 degrees in the first half and −25 degrees in the second half). We combined all trials having the same magnitude of slant and hence there was a total of 360 trials per condition.

Within each block, a psychometric function was measured by varying the slant of the test plane relative to the fixed reference plane. There were eight levels of slant per psychometric function presented in a random order. The texture of the test plane was different on each trial. The texture of the reference plane was different in each block. All observers made judgments for the same stimuli, with a different random order for each observer.

### Control experiments

We ran two control experiments in addition to the main experiment. The first control experiment was a slant-discrimination experiment where the viewing was monocular. Measurements (a total of 160 trials per white-noise level) were only made for the steepest slant, because those stimuli contain the steepest texture gradient, and thus the most reliable monocular information (Knill, 1998; Hillis et al., 2004). The stimuli were constructed using the same rules as those in the main experiment, but observers viewed the stimuli with a patch over the right eye. For all noise levels and slant differences between the test and reference plane used in the actual experiment, the three observers performed at chance.

In the second control experiment, the three observers were asked to discriminate the depth rather than the slant of the test plane relative to the reference plane (see Figure 2C). The reference plane was given a 0 degree slant and was rendered at a distance of 100 cm. The slant of the test plane was fixed at either −44 degrees or 44 degrees so that the near and far edges of the test plane in 3D space were approximately 2 cm in front and behind the reference plane. The test plane was slanted so that the effective disparity pedestal values were similar to those in the main experiment. Consider any pair of test surface points equidistant from the center of the test surface. Assuming that the test plane is perceived to straddle the reference depth, discriminating whether the test plane is closer or further requires determining whether the depth interval to the reference plane for one of the points is greater than or less than that to the other point. On average, this pedestal is about 1 cm. In depth discrimination experiments, it is known that thresholds tend to increase with the magnitude of the disparity pedestal (Blakemore, 1970b; Schumer & Julesz, 1984; Badcock & Schor, 1985; Stevenson, Cormack, Schor, & Tyler, 1992). Thus, for comparing human efficiency in the two tasks it is best to keep the range of disparities similar.

**Figure 3.**
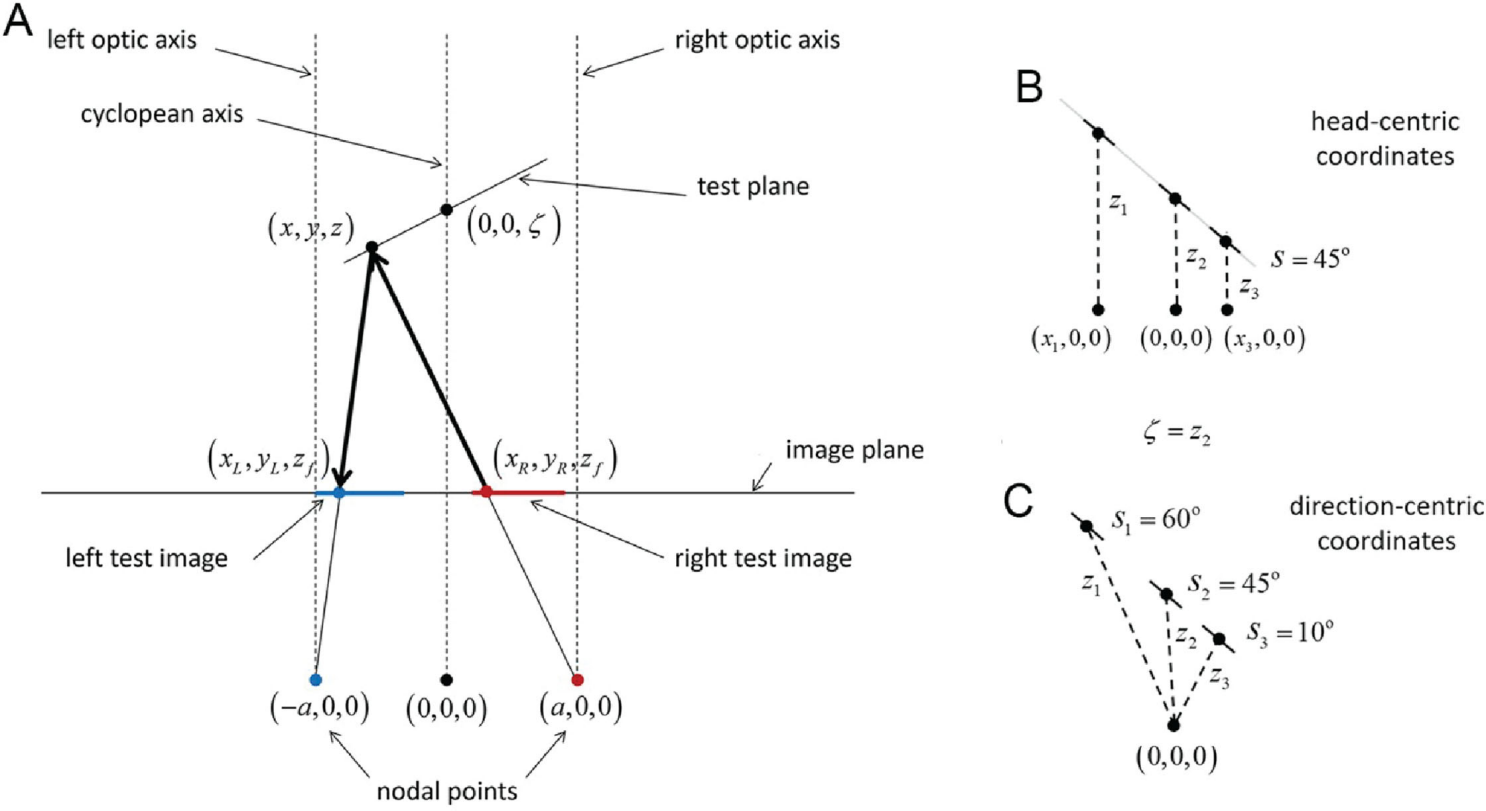
Schematic of the viewing and imaging geometry used for determining ideal and subideal observer performance. (**A**) The test plane is a planar surface whose distance ζ and slant *s* are defined with respect to the cyclopean axis. The left (blue) and right (red) images of the test plane are formed in the cyclopean image plane by perspective projection for nodal points separated by an interocular distance of 2*a*. The ideal observer uses planar matching (PM). For each possible slant and distance of the test plane, the observer predicts the left test image from the right test image (or vice versa) by back projection and then forward projection; for example, the predicted gray level at (*x_L_*,*y_L_*)is the gray level at (*x_R_*,*y_R_*). The estimated slant $\hat{s}$ and distance $\hat{\zeta }$ of the test plane are the values that give the smallest prediction error (Equation 7). (**B**)In head-centric coordinates, all points in the test plane have the same slant. The distance ζ of the test plane is the intercept of the plane, containing the test plane, with the cyclopean axis. The distance *z_i_* of individual points in the test plane varies with location in the image plane. (**C**) In direction-centric coordinates the slant and distance are in general different for different points in the test plane. Model performance is the same for the two coordinate systems, but for simplicity we use head-centric coordinates.

Depth discrimination of the test plane was measured as function of a noise contrast. Each participant completed two sessions, one in increasing order of noise contrast and one in decreasing order. Each session consisted of three pairs of blocks, one pair for each noise contrast. The first block in each pair had half the number of trials, and feedback was provided. There was no feedback in the second block of each pair. There were 80 trials in each no feedback block. The data from the feedback blocks (40 trials) were not used in the analysis. To measure psychometric functions, the depth difference between the reference and test planes varied within the block. There were eight depth difference levels presented in random order. The observers’ task was to report whether the center of the test plane was closer or further than the reference plane. As in the main experiment, different random textures were generated for each trial, and all observers made judgments for the same stimuli. The only difference was that the order of the stimuli was randomly different for each observer.

### Analysis methods

For non-zero reference slants, the response data was the percentage of “more-slanted” responses as a function of the angular difference between test and reference planes. For the zero reference slant (frontoparallel reference plane), the response data was the percentage of “slanted left” responses as a function of test slant. For each combination of reference-slant magnitude and noise contrast, we first merged the psychometric data for the two reference-slant orientations having the same magnitude (e.g. 50 degrees and −50 degrees). The psychometric data for each condition and subject was then fitted with a cumulative Gaussian function using maximum likelihood:

(1) $$\begin{array}{c} p\left(\text{''more}\;\text{slanted''}\right)=\Phi \left(\frac{\Delta s-\beta }{2\sigma }\right)\; \end{array}$$

where Δ*s* is the test slant minus the reference slant, σ is the standard deviation parameter, and β is the bias parameter. The bias parameter corresponds to the 50% point of the psychometric function, and the value of the standard deviation was defined to be the threshold. Note that the discriminability, *d*′, equals Δs/σ. We define threshold to be the value of Δ*s* for which *d*′ = 1.

Not surprisingly, given the well-known individual differences in stereo acuity (Coutant & Westheimer, 1993; Bohr & Read, 2013), there were substantial differences in the overall performance level between the three observers. However, the three pair-wise correlations between participants’ thresholds were quite high (0.967, 0.973, and 0.962). Therefore, to better see the average trends, we scaled the thresholds of two of the observers so that their average threshold was the same as the average threshold of the intermediate performing observer. The scale factor for the more sensitive observer was 0.68 and for the less sensitive observer was 2.01. These same scale factors were also used to scale the biases.

The control experiments were analyzed using the same procedures. However, for the control experiment on depth discrimination, the differences in overall performance between observers were not as large, so the data were averaged without scaling.

## Models

We consider an approximate ideal observer and several suboptimal observers. In all cases, the input to the model is the pair of left and right images produced by the test plane. The left and right images in the test region are described by the following two equations:

(2) $$\begin{array}{c} R\left(x,y\right)=R_{t}\left(x,y\right)+R_{n}\left(x,y\right)\; \end{array}$$

(3) $$\begin{array}{c} L\left(x,y\right)=L_{t}\left(x,y\right)+L_{n}\left(x,y\right)\; \end{array}$$

where *R_t_* and *L_t_* are the images of the sample of 3D texture in the right and left eyes, and *R_n_* and *L_n_* are the independent samples of white noise added to the right and left images.

### Approximate ideal observer: Planar matching

Derivation of the exact ideal observer was not practical for our particular stimuli, because it would require optimal local spatial-frequency filtering to remove frequencies in the uncorrelated noise that do not overlap with the frequencies in the projected texture. We instead optimize a global filter by trial and error, which should give a close approximation to ideal performance. If we let *f*(*x*, *y*) be the optimal filtering kernel in the space domain, then the filtered right and left images are given by the following:

(4) $$\begin{array}{c} R_{f}\left(x,y\right)=\left(f⊗R_{t}\right)\left(x,y\right)+\left(f⊗R_{n}\right)\left(x,y\right)\; \end{array}$$

(5) $$\begin{array}{c} L_{f}\left(x,y\right)=\left(f⊗L_{t}\right)\left(x,y\right)+\left(f⊗L_{n}\right)\left(x,y\right)\; \end{array}$$

where ⊗ represents the operation of two-dimensional cross correlation.

We derive the approximate ideal observer under the assumptions that the imaging geometry is known, and that the 3D surface geometry is described by parameters **θ** = (θ_1_,⋅⋅⋅, θ_*m*_). For planar surfaces, there are three parameters: distance, slant, and tilt. In our experiment, there are only two parameters: distance and slant (the tilt was fixed at zero). The goal of the observer is to estimate the surface geometry from the left and right images. Even after filtering, the added noise remains statistically independent across the two images and is the dominant noise source. Thus, using Bayes rule, the maximum posterior estimate of the surface geometry is given approximately by the following:

(6) $$\;\;\;\;\;\;\;\;\;\hat{\theta }=\underset{\theta }{\arg\max}\left[p\left(\theta \right)\frac{1}{{\left(2\sqrt{\pi }\sigma_{f}\right)}^{n}}\exp\left(-\frac{1}{4\sigma_{f}^{2}}{∥L_{f}-{\hat{L}}_{f}\left(\theta ,R_{f}\right)∥}^{2}\right)\right]$$

In this equation, *p*(**θ**) is the prior over surface geometry, σ_*f*_ is the standard deviation of the filtered samples of white noise, *n* is the number of pixels in the left image, **R**_*f*_ and **L**_*f*_ are the filtered right and left images represented in vector notation, and ${\hat{L}}_{f}\left(\theta ,R_{f}\right)$ is the predicted left image given the right image and a specific surface geometry. The predicted left image is obtained by back projecting the right image to the 3D surface specified by the parameters and then forward projecting to the left eye. Note that one can also project from the left image to the right image (or both ways), but we have found that it makes little difference.

Maximizing the posterior probability is equivalent to minimizing the negative of the log posterior and hence we have the following:

(7) $$\;\;\;\;\;\;\;\;\hat{\theta }=\underset{\theta }{\arg\min}\left[\frac{1}{4\sigma_{f}^{2}}{∥L_{f}-{\hat{L}}_{f}\left(\theta ,R_{f}\right)∥}^{2}+\ln p\left(\theta \right)-n\ln\left(2\sqrt{\pi }\sigma_{f}\right)\right]$$

The squared quantity is the squared error between the left image and the predicted left image. Thus, this equation shows that the approximate ideal observer minimizes the squared error while simultaneously taking into account the prior over surface geometry. Note that when estimated surface geometry matches the true geometry, then the mean difference between the left image and the predicted left image at each location is zero, and the variance at each location is twice the variance of the filtered white noise.

Because of its popularity, we also generated predictions using a normalized-correlation error measure rather than squared error. It performs very similarly, but is computationally much slower. In addition, for a simple absolute disparity estimation task (not slant estimation), we found that these two measures perform similarly and closely approximate the exact ideal observer for that task (Oluk & Geisler, 2020). In other words, using normalized correlation in Equation 7 is also approximately ideal.

The minimization in Equation 7 is over parameters describing the surface geometry, and hence the minimization simultaneously solves the correspondence problem and the translation into an estimation of the surface geometry. On the other hand, the parameters in Equation 7 could also represent an arbitrary mapping between the right and left images without any direct connection to surface geometry. In that case, the estimated parameter values only solve the correspondence problem, and a second step would be required to estimate surface geometry.

For mathematical convenience in generating predictions, we assume a zero vergence angle and an image plane that is perpendicular to the optic axes (standard camera geometry; see Figure 3). However, we note that as long as the positions and orientations of the eyes are known, then the information available for estimating surface slant and distance remains largely invariant. Thus, the performance of the models for the geometry in Figure 3 is quite general. Of course, the specific computations in the models depend on the positions and orientations of the eyes, and on the coordinate system used to describe the projection from scene to image.

The surface geometry in our experiment is described by two parameters the surface slant θ_1_ = *s* and the intercept distance θ_2_ = ζ. Because the tilt was fixed in our experiments, we assumed that the tilt is known. The specific equations for the backward and forward projection are given in the Appendix, for the case where the planar surfaces can have arbitrary slant, tilt, and distance. For present purposes, we assumed a uniform prior over slant (±70 degrees) and over intercept distance (100 cm ± 1 cm), which covers the full range of possibilities in the main experiment.

In our experiment, the display was designed so that the image of the test plane in the right eye always had the same width (2.23 degrees), which is assumed to be known by the model observers. To further simplify computations, the predictions were generated for a fixed height region (2.12 degrees) in the right-eye image, which corresponds to the minimum height produced in the experiments. This leaves out approximately 5% of the informative pixels, but has a minor effect on the predictions.

Recall that the test-plane and reference-plane textures were a sum of random sine waves having a maximum spatial frequency of 3 cpd, before rotation about the vertical axis. The image spatial frequencies in the test and reference planes can be higher because of the surface slant. In the experiment, uncorrelated white noise was added to the left- and right-eye test-plane regions. As mentioned earlier, the ideal observer must prefilter the stimuli before comparing the left and right eye images; that is, it must filter out spatial frequencies in the added white noise that do not overlap with frequencies present in the signal. To approximate the optimal prefiltering, we carried out a preliminary analysis where we measured PM performance for various values of the cutoff frequency of a low-pass filter that ramped to zero over a span of 1 cpd. This was done separately for each level of uncorrelated noise. We found that the optimal cutoff frequencies for the 5%, 17.5%, and 34% noise contrasts are 16, 8, and 5 cpd, respectively. Variation in these cutoff frequencies by 1 to 2 cpd had little effect on performance. We used this same prefiltering for all model observers.

**Figure 4.**
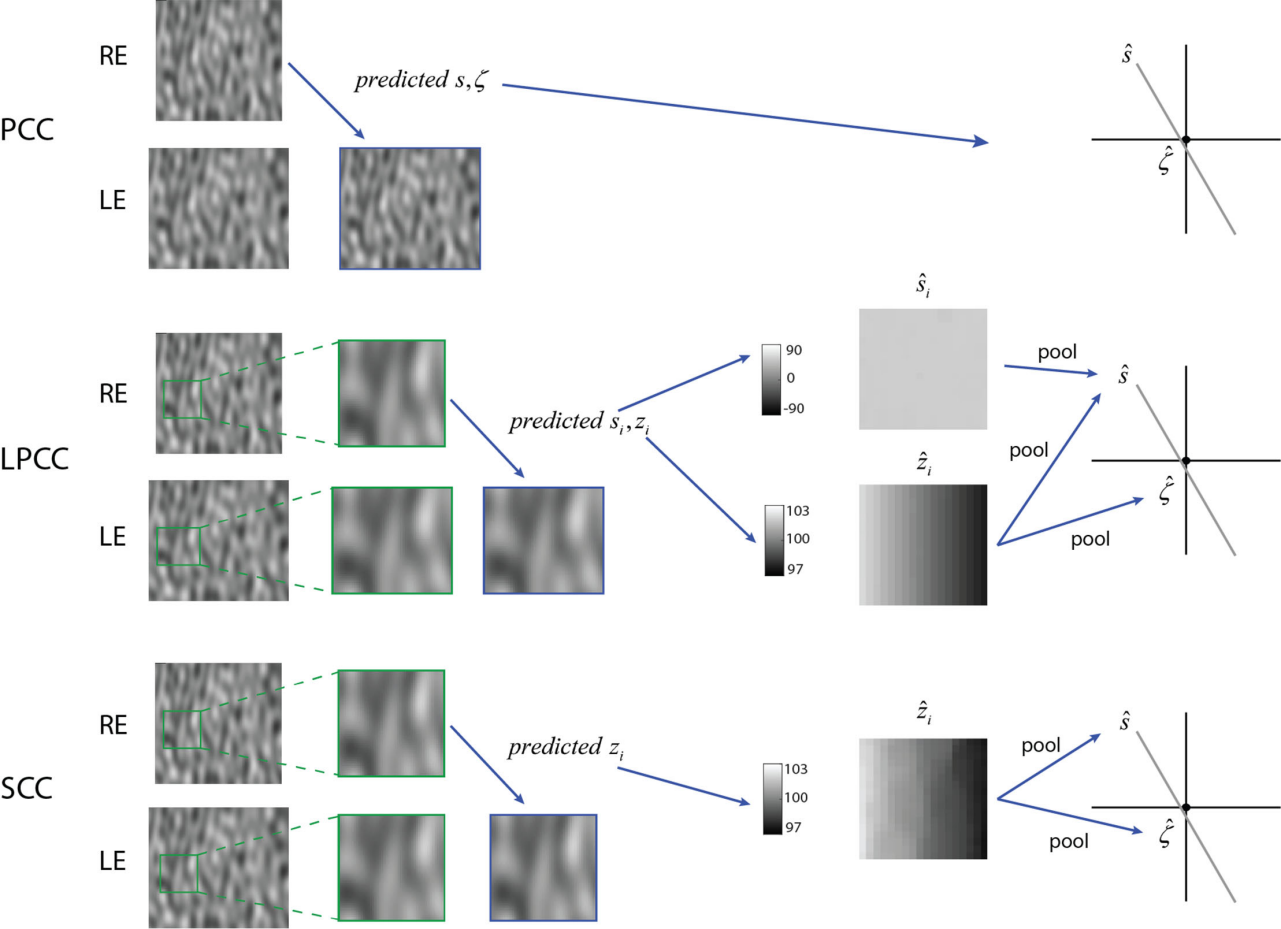
Illustration of computations in three model observers (PM, LPM, and LFM), for the case where there is no uncorrelated white noise added to the left and right images. In the experiments, the stimuli always included uncorrelated white noise (see Figure 2).

The first two images in the upper row of Figure 4 show example right and left images when the slant is +60 degrees and the intercept distance is 99 cm. When the estimated slant and distance are correct, then the predicted image closely matches the left eye image. Here, the estimate is very accurate because there is no added uncorrelated noise. We note that because the reference plane contained no uncorrelated noise, its parameters were always estimated with very high precision. This was also true for the other models. Thus, we were able to simply assume the slant and distance of the reference plane were known.

### Local planar matching

It is not biologically plausible that PM is computed in one step over the entire test region (especially if the test region is larger than the current 2.13 degrees width). Thus, we also consider a suboptimal version where PM is computed over square right-eye patches of some given width *w*. The computations for each patch are basically the same as described above, except now Equation 7 is applied to local patches within the test region.

**Figure 5.**
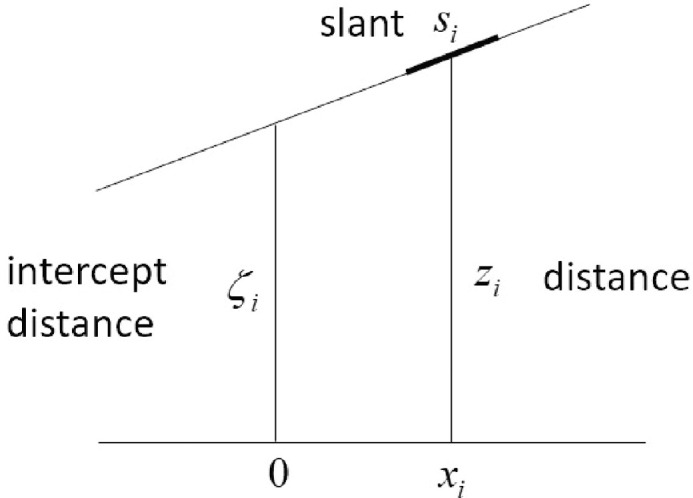
Local planar matching. Illustration of the difference between intercept distance and distance. Local planar matching uses estimates of local slant and distance. Local frontoparallel matching uses estimates of local distance assuming the local slant is zero.

The only important difference is that we express the surface geometry in terms of the slant *s_i_* and distance of the patch *z_i_*, rather than the slant *s_i_* and intercept distance ζ_*i*_ (see Figure 5). In other words, in Equation 7, we take **θ**_*i*_ = (*s_i_*,*z_i_*) rather than **θ**_*i*_ = (*s_i_*,ζ_*i*_). The reason is that the estimates of slant and intercept distance become more correlated the larger the horizontal distance (*x_i_*) of the patch from the cyclopean axis (i.e. changes in the estimate of slant cause changes in the estimate of intercept distances). On the other hand, slant and distance are nearly statistically independent everywhere. The relationship between the intercept distance and distance of the patch is given by ζ_*i*_ = *z_i_* − *x_i_* tan *s_i_*, which can be substituted into the equations in the Appendix to express the backward and forward projections in terms of slant and distance.

Because the local slant and distance estimates are relatively independent, it is then possible to obtain two independent estimates of the global slant, one by pooling the local distance estimates as follows:

(8) $$\left({\hat{s}}_{z},{\hat{\zeta }}_{z}\right)=\underset{s,\zeta }{\arg\min}\;\;\sum_{i=1}^{n}{\left[{\hat{z}}_{i}-\left(\zeta +x_{i}\tan s\right)\right]}^{2}$$

and one by pooling the local slant estimates as follows:

(9) $${\hat{s}}_{s}=\underset{s}{\arg\min}\;\;\sum_{i=1}^{n}{\left[{\hat{s}}_{i}-s\right]}^{2}$$

Finally, the two slant estimates can be combined using reliability-weighted cue combination, which is optimal for bias-corrected statistically-independent cues (Cochran, 1937; Clark & Yuille, 1990; Oruc, Maloney, & Landy, 2003):

(10) $$\hat{s}=\frac{r_{z}{\hat{s}}_{z}+r_{s}{\hat{s}}_{s}}{r_{z}+r_{s}}$$

where *r_z_* and *r_s_* are the reliabilities of the two estimates. To measure the reliability of the slant estimates for each cue, we computed slant estimates (for many test patches) separately for each reference slant. From these estimates we then computed the bias in the slant estimates for each reference slant. Finally, the reliability of the estimates was determined from the standard deviation of a cumulative Gaussian fit to bias-corrected slant estimates, as a function of test-patch slant. The reliability of the slant estimates was taken to be one over the square of this standard deviation (i.e. the reciprocal variance).

The middle row in Figure 4 illustrates the LPM observer. For each square patch in the right image, an estimate of the left image patch is generated. When the assumed slant and distance are correct then the predicted left image patch will closely match the left image patch, at least when the uncorrelated noise is absent (as it is in the Figure). Thus, the predicted slant for each patch will be the same and will be very close to correct, as will the predicted distance. Finally, pooling the slant and distance maps gives an estimate of the slant of the whole surface and pooling the distance map gives an estimate of the intercept distance of the whole surface.

### Local frontoparallel matching

The simplest model observer considered here is the local frontoparallel matching model, which is a special case of the local PM model where the local slant is assumed to be zero (i.e. Equation 7) with **θ** = (0, *z_i_*), which for the geometry in Figures 3 and 5 is equivalent to horizontally translating each patch in the right eye to find the best match in the left eye to obtain an estimated disparity, and then computing the estimated distance given the separation between the eyes. The estimated slant and intercept distance of the test plane are then obtained by applying Equation 8 to the set of estimated distances. These computations are illustrated in the bottom row of Figure 4. Note that there are errors in the estimated local distances (visible as the lumpiness in the ${\hat{z}}_{i}$ map). These failures to perfectly solve the local correspondence problem occur because of the model's assumption that the local slant is zero.

### Estimation noise and overall efficiency scalar

The PM, LPM, and LFM models above have no free parameters. We also considered suboptimal models with two other limitations, each specified by a free parameter: (i) a fixed level of internal noise σ_0_ added to the slant estimates, and (ii) an overall efficiency scale factor ε that scales down all discriminability (*d*′) values. Specifically, the discriminability of the model observers with these two free parameters is given by the following:

(11) $$d^{'}\left(\Delta s\right)=ɛ\frac{\Delta s}{\sqrt{\sigma^{2}+\sigma_{0}^{2}}}$$

where Δ*s* is the mean difference in estimated slant between the test and reference plane for a model observer with no free parameters, and σ is the standard deviation of these estimated slant differences. Note that the efficiency scalar could correspond to scaling down the numerator, scaling up the denominator, or some combination. Given threshold is defined as a discriminability of 1.0, the slant-discrimination threshold of the model observers is given by the following:

(12) $$\Delta s_{t}=\frac{1}{ɛ}\sqrt{\sigma^{2}+\sigma_{0}^{2}}$$

Other obvious sources of intrinsic noise are photon noise and neural noise in the retina and LGN. This noise can be modeled as statistically independent (uncorrelated) across the eyes, and thus has a similar effect to the uncorrelated noise added to the left and right images. However, its effect is similar to that of the estimation noise so we do not show the predictions here.

### Model predictions

The predicted slant-discrimination thresholds were obtained by simulating the model-observer estimates of the slant difference between test and reference planes. On each trial, a model observer returns an estimate of the slant difference between the test plane and reference plane (which was assumed to be known). To obtain predicted thresholds, slant-difference estimates were generated for the specific test and reference plane slants presented to the human observers. For each condition, we computed the mean and the standard deviation of the slant-difference estimates of the model being fitted. From these means and standard deviations, we computed the error rates predicted given any assumed values of the internal noise and scale parameters. The internal-noise and efficiency-scalar parameters were estimated by the maximizing likelihood. The predicted thresholds are calculated from fitted error rates. An equivalent procedure was used for estimating the predicted depth-discrimination thresholds, except only the parameters ζ or *z* were varied. For more details about the model predictions see https://github.com/CanOluk/Stereo-Slant-Discrimination.

### Alternate versions and interpretations of model observers

All the models described above assume that the absolute slant and distance of the reference plane are known. This is justified because all of our model observers estimate the slant and distance of the reference plane (which has no added noise) with high precision. All the models also assume that slant discrimination is performed by estimating the absolute slant and distance of the test plane and comparing the estimated absolute slant with the known absolute slant of the reference plane. This approach was taken for simplicity. However, largely equivalent predictions would be obtained by directly using the estimated location and pattern disparities from LPM observer, or estimated location disparities from LFM observer, to compute the difference in disparity and disparity gradients for test and reference planes, without ever estimating absolute slants and distances. As long as the absolute distances of the of the test and reference plane are not drastically different (which they were not in the current study), then there is no need to directly estimate absolute slant and distance. Furthermore, models that use only disparity and disparity gradients perform similarly even when there are modest levels of eye pose uncertainty (see Discussion).

All the model observers described above assume that the properties of the 3D scene (distance or distance and surface orientation) are estimated by directly searching for the scene properties that best predict the test image in one eye from that in the other eye using backward and forward projection (see Figure 4). However, the matching computations in the LPM and LFM models can also be regarded as different methods for estimating the location disparities between pixels in the left and right images. The set of possible backward and forward projections define the set of transformations (location and pattern changes) of a local image patch in one eye that are explored to find the best matching local image patch in the other eye. Thus, the difference in the center-pixel locations of the best-matching patches is the estimate of the disparity at the center of each patch (there is a patch centered on each pixel). These location disparities, and knowledge of the priors over scene geometry, represent all the binocular-disparity information used by LFM observer, and most of the binocular-disparity information used by the LPM observer. The additional information used by the LPM observer is the specific pattern disparities between the best matching patches. However, the pattern disparities provide a somewhat less reliable slant estimate than the location disparities (except when patch size gets quite large). Thus, another version of the LPM observer would be one where only the location disparities are used. This version of the LPM observer still performs substantially better than the LFM observer for planar surfaces, especially for larger slants and larger sizes of the local patches used in the matching. It also makes very similar predictions (not shown here) to the full LPM observer.

Finally, we note that none of the models are meant to represent biologically plausible computations. Rather they are meant to represent computational principles that exploit the available disparity information in different ways. Nonetheless, at least for the LFM and LPM observers, these computational principles could be implemented in biologically plausible ways (see Discussion).

## Results

### Slant discrimination

**Figure 6.**
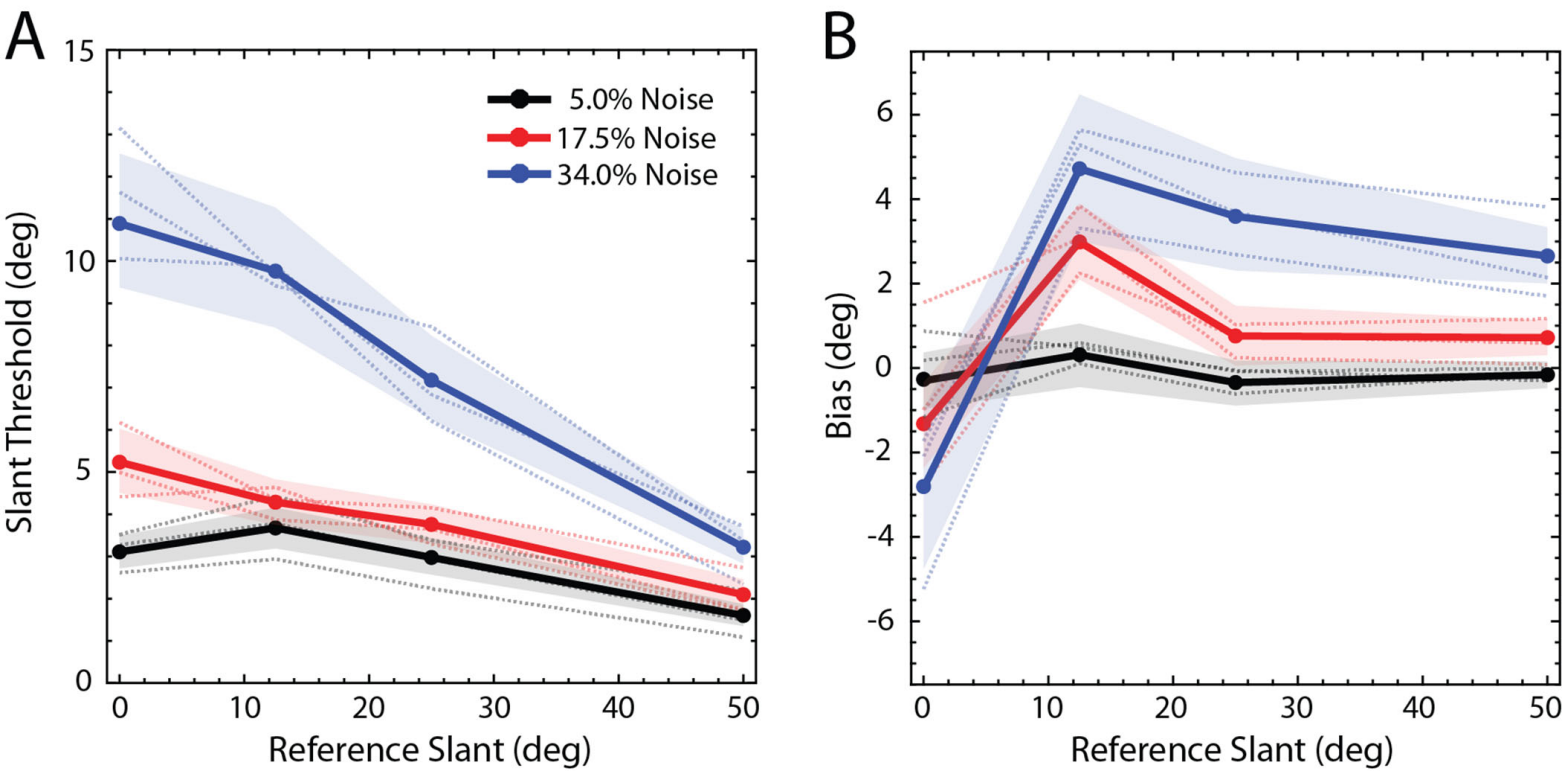
The results of the slant experiment. (**A**) Mean thresholds are shown with solid lines and individual thresholds are shown with dotted lines. Ninety-five percent confidence intervals are shown as shaded regions. They were computed from the average psychometric functions by resampling each point 2000 times assuming that the number of “more slanted” responses is binomially distributed. For each resampling, the threshold (and bias) was recomputed to obtain its estimated distribution. (**B**) Mean biases are shown with solid lines and individual's biases are shown with dotted lines. The individual subjects have similar shaped threshold curves, but they differ in overall sensitivity. In this plot, the most sensitive and the least sensitive observer's thresholds were each scaled by a single factor to best match the medium sensitive observer's thresholds. The biases were scaled by the same factors. The two scale factors are 0.68 and 2.01.

**Figure A1.**
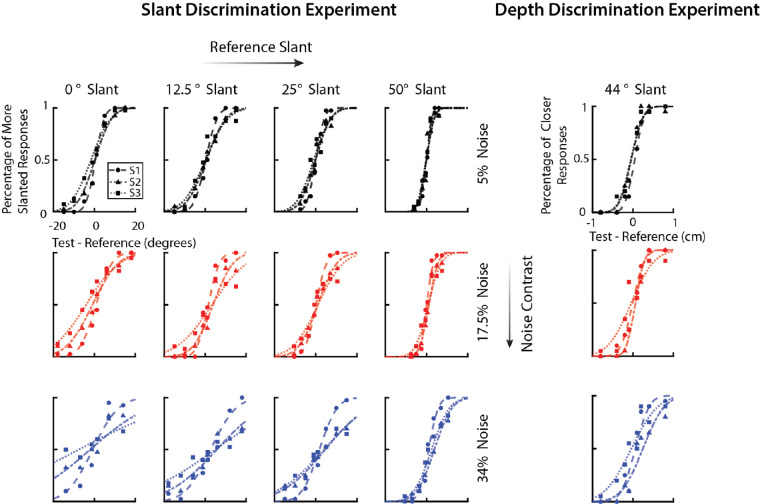
Psychometric functions from slant and depth discrimination experiments, for the three observers (S1, S2, and S3). The smooth curves were fitted by maximum likelihood and were used to determine the thresholds and biases reported in Figures 6 and 11.

Figure 6A shows the scaled slant discrimination thresholds of the three observers (dotted curves) and their average thresholds (solid curves). (The psychometric functions of the individual observers are given in the Appendix, see Figure A1.) As can be seen, thresholds increased with the noise contrast (plot color) and decreased with the absolute reference slant (increasing along the abscissa). As noted earlier, the individual human observers exhibited similar patterns of slant-discrimination thresholds and biases, but the thresholds differed substantially in overall sensitivity. To better compare the threshold and bias patterns, we separately scaled the thresholds of the most sensitive and the least sensitive observer by an overall factor to best match (in squared error) the thresholds of the medium-sensitive observer; the biases were scaled by the same factors (see Methods).

Figure 6B shows the biases. When the RMS contrast of the noise was 34% (blue), the frontoparallel test plane (slant of 0 degrees) was perceived as if its right edge was slightly behind the left edge (95% confidence interval: −5.33 to −1.04 degrees), and the slanted test planes were perceived as slightly more frontoparallel than their true slant (95% confidence intervals of 3 degrees to 6.6 degrees, 2.3 degrees to 5.1 degrees, and 1.9 degrees to 3.3 degrees for reference slants of 12.5 degrees, 25 degrees, and 50 degrees, respectively). Similar, but weaker, frontoparallel biases were found for 12.5 degrees and 50 degrees reference slants when the RMS contrast of the noise was 17.5% (95% confidence intervals of 2.2 degrees to 4 degrees and 0.2 degrees to 1 degree, respectively). A potential explanation for the frontoparallel bias is that the visual system exploits knowledge of the prior probability distribution of slants in natural scenes, which have been found to peak at a slant of zero (Burge et al., 2016). When the uncorrelated noise levels are high, the slant estimates necessarily become less reliable. When this occurs, it is rational for the visual system to place more weight on the prior probability distribution, causing a greater bias toward zero slant (e.g. see Weiss, Simoncelli, & Adelson, 2002).

**Figure 7.**
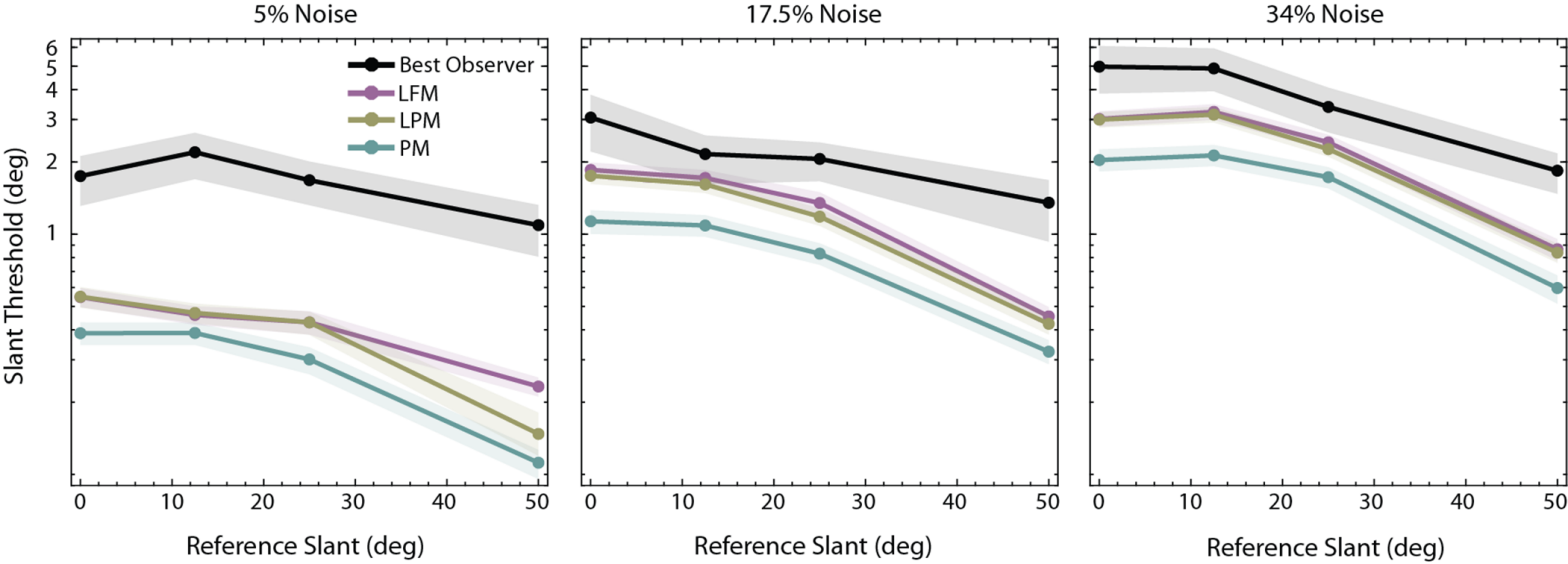
The absolute slant discrimination thresholds of three model observers and the best-performing human observer. The planar matching (PM) model is near optimal and has no free parameters. The local frontoparallel matching (LFM) model is suboptimal. The only parameter is the matching patch width (*w*), which was picked to give the best overall absolute performance (*w* = 0.5 degrees). The local planar matching (LPM) model is also suboptimal. Shown here is the performance with the same patch width as the best performing LFM model (*w* = 0.5 degrees). Shaded regions correspond to 95% bootstrapped confidence intervals. Note that in this figure (unlike Figure 6) the thresholds are plotted on a logarithmic scale.

Figure 7 shows the absolute thresholds for the first family of model observers along with the most sensitive human observer on a log y-axis. The thresholds of the three model observers are given by the colored symbols. The PM model has no free parameters. The LPM and LFM models each had a single free parameter: the patch width *w*. The value of this parameter was set so as to maximize the performance of the LFM model. The value of the patch-width parameter in the LPM model was identical to the patch width in the best-performing LFM observer. All three models outperform the best-performing human participant in the experiment (black symbols). As expected, the thresholds of the PM observer (blue symbols) are lowest in all conditions. The performance of the LPM model is similar to the LFM model; however, if the patch width used for the LPM observer is made larger, its performance improves, and (of course) asymptotes to the performance of the PM observer.

**Figure 8.**
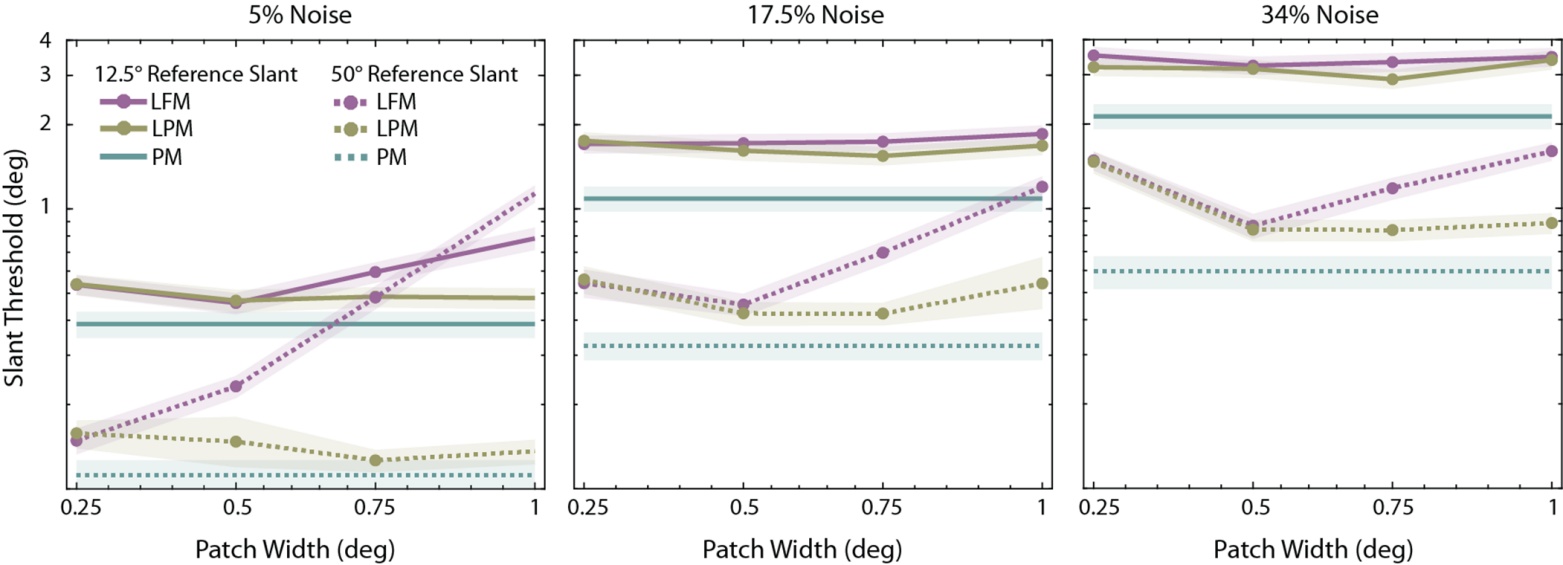
The change in absolute slant discrimination thresholds of LPM and LFM model observers as a function of patch width. The patch width for the PM model was the full size of the target in the right eye (2.2 degrees). The solid lines correspond to 12.5 degrees reference slant and dashed lines correspond to 50 degrees reference slant. Shaded regions correspond to 95% bootstrapped confidence intervals. Note that the thresholds are plotted on a logarithmic scale.

Figure 8 shows the thresholds of LPM and LFM models for different patch width values when the reference slant is either 12.5 degrees or 50 degrees (solid and dashed lines). As patch width increases, the LFM model thresholds become systematically worse than those of the LPM and PM models, whereas the LPM model thresholds either improve slightly or remain stable. The LPM model is therefore more robust to changes in patch width. As expected, the LFM model performs particularly poorly when the reference slant is high because it is the condition when the implicit assumption of frontoparallel surfaces is most inaccurate. Last, the difference between LPM and LFM is smaller when the external noise is high, probably because in high noise the effect of external noise tends to exceed the effect of the differences in specific computations.

**Figure 9.**
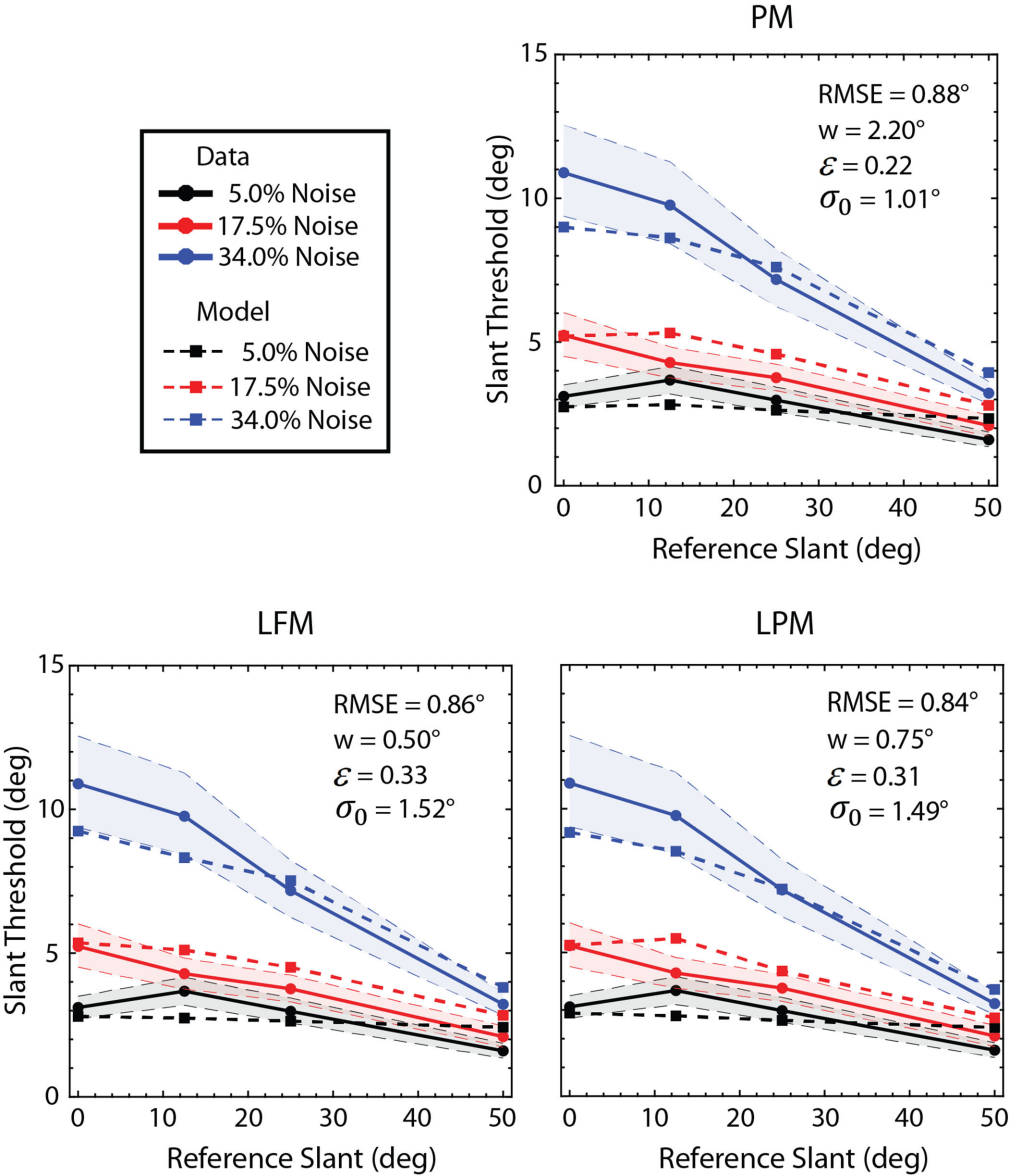
Model fits. For each model observer, the patch width (*w*), scale factor (ε), and estimation noise standard deviation (σ_0_) were estimated by maximizing the likelihood of the data. The patch width for the PM model was the full size of the target in the right eye (2.2 degrees). Although the parameters were estimated by maximizing likelihood, the goodness-of-fit measure shown in the figure is the RMSE, which is more intuitive.

Figure 9 shows the maximum-likelihood fits of the three models (dashed curves) to the average thresholds in Figure 6A, when the patch width (*w*), estimation-noise standard deviation (σ_0_) and overall efficiency scale factor (ε) were allowed to vary (only σ_0_ and ε were allowed to vary for the PM model). Note that although the fits were obtained by maximizing likelihood, we report the root-mean-squared error (RMSE) in the figure because it is more intuitive. The predicted rate in fall-off in the thresholds with reference slant is similar to that in the human observers. Note that the best fitting values of σ_0_ are quite small, on the order of 1 degree to 1.5 degrees, and hence are in a plausible range. Surprisingly, the predictions are about equally good for the three models, so the data are not sufficient to differentiate between the models.

**Figure 10.**
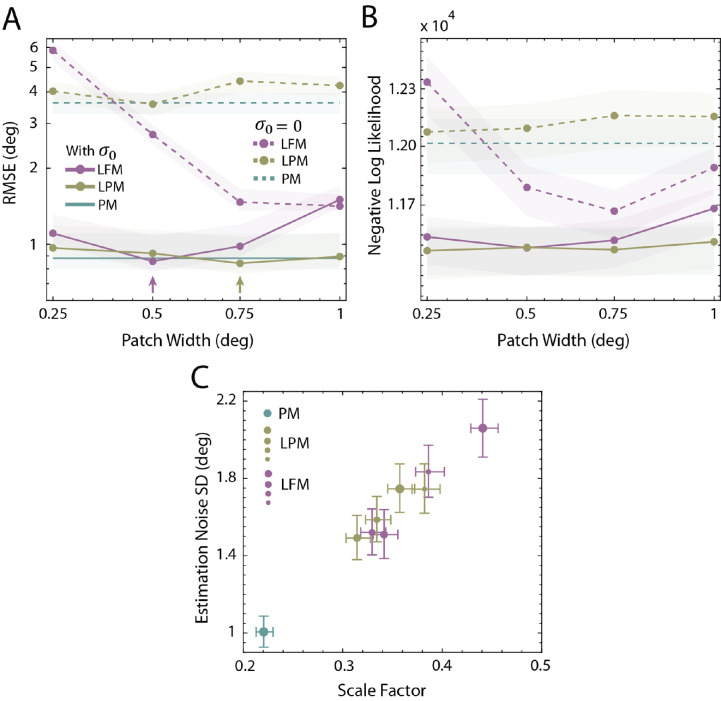
Results of the model fitting procedure. (**A**) Root-mean squared error (RMSE) for the three models as a function of patch width. The arrows indicate the smallest RMSE for the LFM and LPM models. The parameters were actually estimated by maximizing likelihood (minimizing negative log likelihood). (**B**) Negative log likelihood for the three models as a function of patch width. For the LFM model the RMSE and negative log likelihood are minimal at the same patch width. For the LPM model the RMSE is minimal at 0.75 but the negative log likelihood is minimal at 0.25. (**C**) Estimated scale factor and estimation-noise standard deviation for the three models. For the LPM and LFM models, increasing symbol size represents increasing patch width (0.25, 0.5, 0.75, and 1 visual degree, respectively). Error bars are 68% bootstrapped confidence intervals.

Figures 10A and 10B are plots of goodness-of-fit measures (RMSE and negative log-likelihood, respectively) as a function of patch width. The arrows in Figure 10A indicate the patch widths of the predictions shown in Figure 9. They correspond to the best fits in terms of RMSE. Both plots show that including the estimation-noise parameter greatly improves model predictions (shaded regions are 68% confidence intervals). Figure 10C shows the maximum-likelihood-parameter estimates of each model (symbol color) for each patch width (symbol size). The estimated noise and scalar parameters are largest for the LFM model, smaller for the LPM model, and smallest for the PM model.

In a control experiment, participants performed the slant discrimination experiment with the right eye covered to determine the usefulness of monocular cues. The tested slant was 50 degrees because this was the largest slant magnitude in the main experiment and because perspective (monocular) cues are strongest in this case (Knill, 1998). Psychometric functions were measured for all three noise levels. It was not possible to estimate thresholds, because the slopes of the psychometric functions were always near zero. We conclude that the human slant discrimination thresholds were based entirely on binocular (stereo) cues.

### Depth discrimination

**Figure 11.**
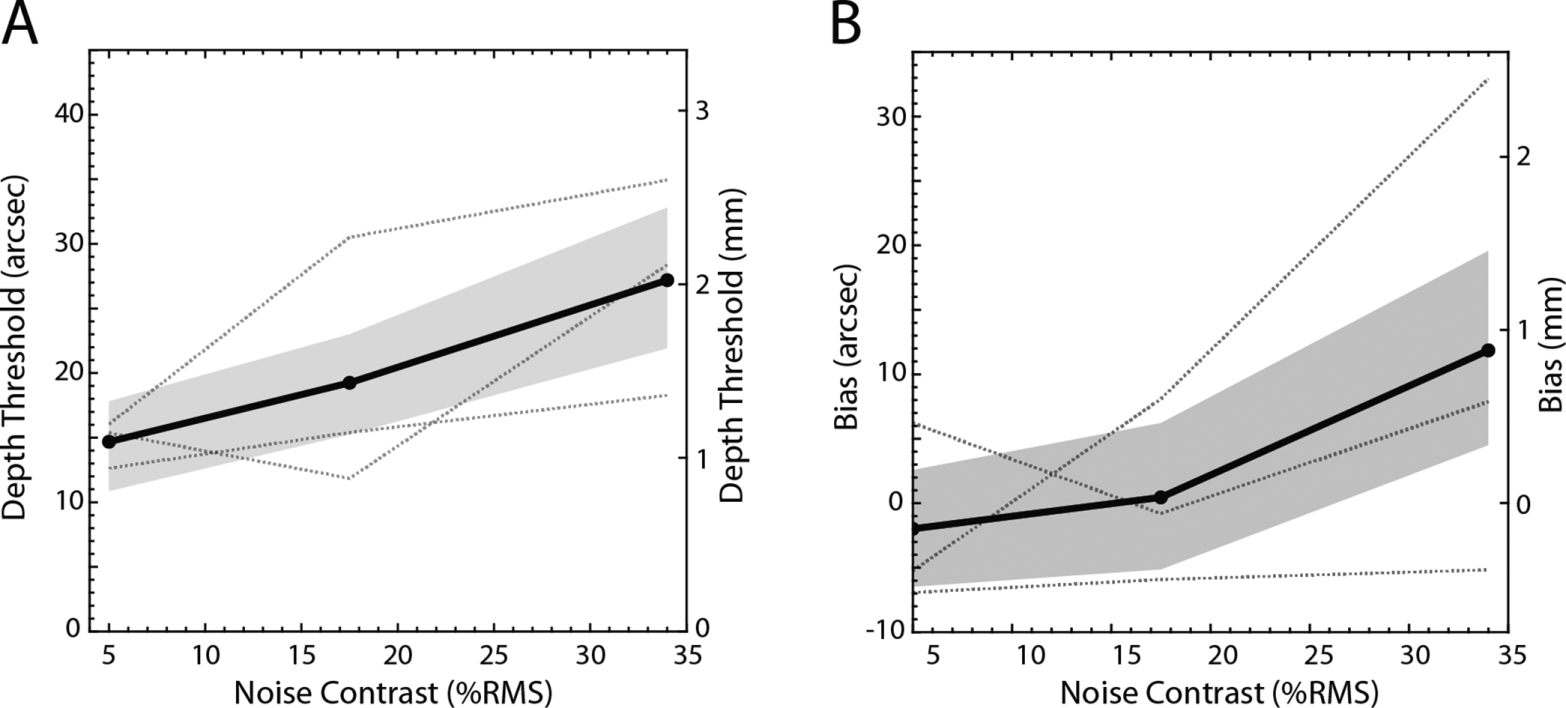
The results of the depth experiment. (**A**) Mean thresholds are shown with solid lines and individual thresholds are shown with dotted lines. Ninety-five percent confidence intervals are shown as shaded regions (see Methods for details). (**B**) Mean biases are shown with solid lines and individual's biases are shown with dotted lines. Ninety-five confidence intervals are shown as shaded regions (see Methods for details).

Figure 11A shows the depth discrimination thresholds of the three observers (dotted curves) and their average (solid curve), and Figure 11B shows the biases. The individual differences were less in this experiment than in the slant-discrimination experiment, and hence there is no scaling of the thresholds for the least and most sensitive participants. As can be seen, human thresholds increase with noise contrast. When the noise is highest (34% RMS), there is also a small bias in two of the subjects to see the test plane slightly farther than the reference plane (the 95% confidence interval of the average bias is 3.9 to 19.8 arc sec).

Similar to the slant experiment, the depth discrimination thresholds of the optimal PM model are better than those of the LPM and LFM models (1.3 seconds of arc better on average). Thresholds of LPM and LFM models are generally similar but the performance of LPM is considerably better than LFM in the low noise condition for 0.75 degrees and 1 degree patch widths (0.5 and 1.5 seconds of arc better, respectively).

The maximum likelihood fits of the three models to the data revealed similar results to the slant experiment. Without the estimation noise parameter, the three models do not fit the thresholds well (RMSE = around 30 seconds of arc, or 2.2 mm). When the estimation noise parameter had a standard deviation of 5 to 7 seconds of arc (0.3–0.5 mm), all three observer models fit the thresholds quite well (RMSE = approximately 1 second of arc or 0.07 mm).

**Figure 12.**
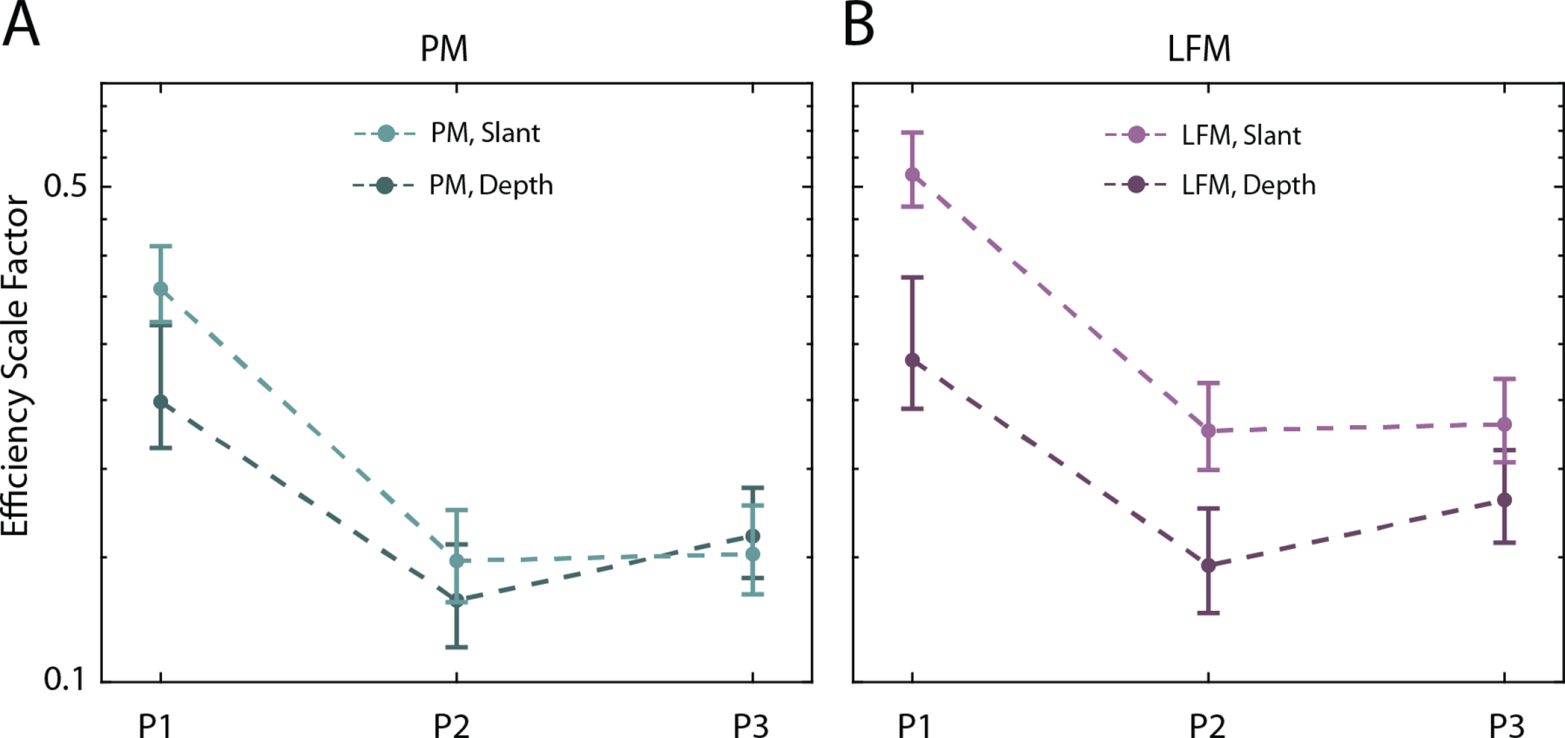
The efficiency scale factors estimated from two different experiments. (**A**) Using the PM model, for three participants, scale factors are estimated by maximizing the likelihood of the data of the highest noise condition either for the slant (50-degree reference slant condition) or the depth experiment. The 68% bootstrapped confidence intervals are shown with error bars. (**B**) Using the LFM model observer, for three participants, scale factors are estimated by maximizing the likelihood of the data of the highest noise condition either for the slant (50-degree reference slant condition) or the depth experiment. The 68% bootstrapped confidence intervals are shown with error bars.

Recall that in the depth experiment the slant of the reference plane was zero, but the test plane was slanted at 44 degrees. This was done so that the range of disparity values was similar to those in the main experiment, making it easier to compare human efficiency for slant and depth discrimination. The PM observer is approximately ideal for both slant and depth discrimination. Thus, it is possible to compare absolute efficiencies in the two tasks (no internal noise in the model observers). The efficiency scale factors for aligning the PM thresholds with each participant's thresholds in high noise (with reference slant of 50 degrees for slant discrimination) are shown in Figure 12A. We only considered the high-noise condition because sampling effects, and machine accuracy limits, made it difficult to generate accurate predictions for depth discrimination at lower noise levels. For two participants (P1 and P2), there is a trend for human efficiency to be higher in the slant discrimination experiment.

It is also possible to estimate the efficiency scale factors for the LFM model. For all participants, the efficiency is higher in the slant experiment, and the confidence intervals do not overlap (Figure 12B). Overall, humans appear to be more efficient (relative to ideal) at estimating relative surface slant than relative surface distance.

## Discussion

We derived the approximate binocular ideal observer for discrimination of the 3D orientation and distance of textured planar surfaces viewed in the presence of additive white noise that is uncorrelated across the two eyes. This PM observer first filters the left and right images, based on the known frequency content of the texture, to remove irrelevant frequency components due to the white noise. Using projective geometry, the PM observer then generates a predicted left image from the right image (or vice versa) for each possible 3D orientation and distance of the test plane. The estimated surface orientation and distance are the values that make the most accurate prediction of the left image (i.e. the smallest squared error, given the prior). We also considered two suboptimal observers that also pre-filter the left and right images. The LPM observer uses the same fundamental computations as the PM observer, but it makes multiple local estimates of the 3D orientation and distance in local image regions, and then combines those local estimates to obtain a single estimate of the 3D orientation and distance of the entire test plane. The LFM observer uses projective geometry to estimate the distance of local image regions under the assumption that the local surface slant is zero. It then combines those estimates to obtain an estimate of the 3D orientation and distance of the test plane. Each of the three model observers has two free parameters: an overall efficiency parameter and a parameter representing a fixed level of internal estimation noise. All the model observers assumed standard camera geometry in cartesian coordinates, but the predictions would be the same for other coordinate systems and eye orientations, as long as they are known.

In terms of absolute performance, the PM observer performs substantially better than the LPM and LFM observers, because it optimally combines all the disparity information over the approximately 2 degrees × 2 degrees test plane. The LPM observer performs better than the LFM observer, especially for larger patch sizes and slants, and it is also less affected by patch size and surface slant (it is more robust) than the LFM observer.

We compared the performance of these model observers with human observers in a stereo slant-discrimination experiment. Human thresholds were measured as a function of the reference slant and the noise contrast of separate samples of white noise added to the left eye and right eye images. Human thresholds decreased with the slant of the reference plane and increased with the level of uncorrelated noise. The pattern of thresholds was consistent across the three human observers. In control experiments, we found that the human thresholds were based on stereo cues alone, and that there is a trend for humans to be more efficient at slant discrimination than depth discrimination. All three models were able to predict the measured thresholds with approximately equal quantitative accuracy.

### Limitations and extensions of model observers

An obvious omission from the model observers is the intrinsic noise that occurs prior to binocular interaction. As mentioned earlier, it has a similar effect to the uncorrelated extrinsic noise added to the left and right images. In addition, its effect on predictions for the current experiments is similar to that of the estimation noise and so, to keep the number of free parameters down, it was not included. However, more plausible models should include both early uncorrelated noise and late estimation noise. We also note that the existence of intrinsic uncorrelated noise implies that the prefiltering step in the model observers is always useful in real visual systems, even when there is no extrinsic uncorrelated noise.

A limitation of the model observers is that they assume eye pose is known exactly. As mentioned earlier, this was done to simplify the implementation of all the model observers. However, it is certain that eye pose cannot be known exactly in the human visual system. Eye pose appears to be estimated by a combination of oculomotor cues (probably efference copy signals) and image cues (e.g. see Backus et al., 1999). One way to generalize the current models would be to assume that the average eye pose is known, but the actual pose randomly varies around that average. This vergence and version noise would inject noise into the predicted images (see Figure 4) and hence into the estimated slant and distance. Alternatively, the visual system could have knowledge of its own eye pose estimation noise and accept matches within the uncertainty range determined by the noise. The effect of eye-pose uncertainty would apply to all the model observers, and hence, as long as the eye-pose estimation noise is modest, the pattern of thresholds and the relative performance of the model observers should remain about the same.

All the model observers directly compute the absolute slant and distance of the test surface and reference surfaces. However, as noted earlier (see Methods), nearly equivalent LPM and LFM observers could be obtained by directly using the estimated location and pattern disparities from LPM observer, or estimated location disparities from LFM observer, to compute the differences in disparity and disparity gradients for test and reference planes, without ever estimating absolute slants and distances. The performance of these models would be more robust to eye-pose uncertainty. Therefore, if the human visual system is performing the slant discrimination task using disparity and disparity gradient computations (Wardle & Gillam, 2016), then it will also be relatively robust to eye-pose uncertainty. If so, then our modeling assumption of known eye pose has relatively little effect on the predictions for our experiments.

Human performance varies depending on the proximity of the test and reference surfaces in space and time (Gillam, Flagg, & Finlay, 1984; van Ee, Banks, & Backus, 1999*)*. Specifically, humans are most precise at computing differences in the slant and distance of surfaces (which is why we used the stimuli illustrated in Figure 2). What the current models do not predict are changes in performance with increased separation in space and time between the test and reference. To make plausible predictions for such experiments would require including other factors, such as memory limitations, disparity contrast mechanisms, and reduced spatial resolution in the periphery. The importance of the proximity of the test and reference surfaces may explain the surprising observation that humans appear to be more efficient at slant discrimination than distance discrimination (see Figure 12). In the slant task, the depth information (relative to the reference plane) is concentrated near the edges of the test plane; whereas, for depth discrimination the relative depth information is uniformly distributed across the test plane. If humans are better able to integrate information near the reference plane, then their efficiency (relative to the model observers) should be higher in the slant task.

### Solving the binocular correspondence problem

The three model observers solve the correspondence problem in different ways. The PM observer finds the distortion and translation that minimizes the difference between the entire left and right test images, given planar surfaces. The LPM observer finds, for each pixel in one eye, the distortion and translation that minimizes the difference between the patch surrounding that pixel and one of the potential patches in the other eye, given planar surfaces. The LFM observer finds, for each pixel in one eye, the translation that minimizes the difference between the patch surrounding that pixel and one of the potential patches in the other eye.

The LFM and LPM observers are both biologically plausible in that they involve local binocular comparisons that could potentially be implemented with binocular receptive fields. The LFM observer is consistent with the hypothesis that early receptive fields are explicitly coding binocular differences in horizontal phase and location (location disparities), and that these are integrated in later areas to yield receptive fields sensitive to distance and surface orientation.

**Figure 13.**
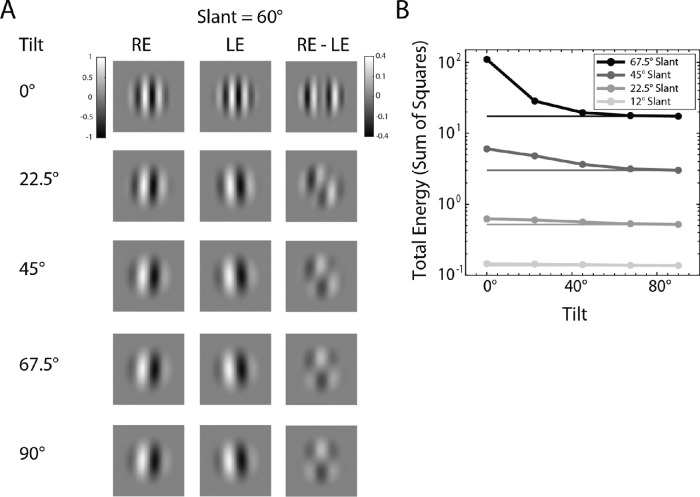
Hypothetical binocular receptive fields tuned to surface orientation and distance. (**A**) Examples: right eye receptive field (RE), left eye receptive field (LE), difference between right and left eye receptive fields. (Note that the gray scale for differences is 40% of the gray scale for the receptive fields.) (**B**) Energy of the difference between right and left receptive fields as a function of tilt, for several different slants.

**Figure A3.**
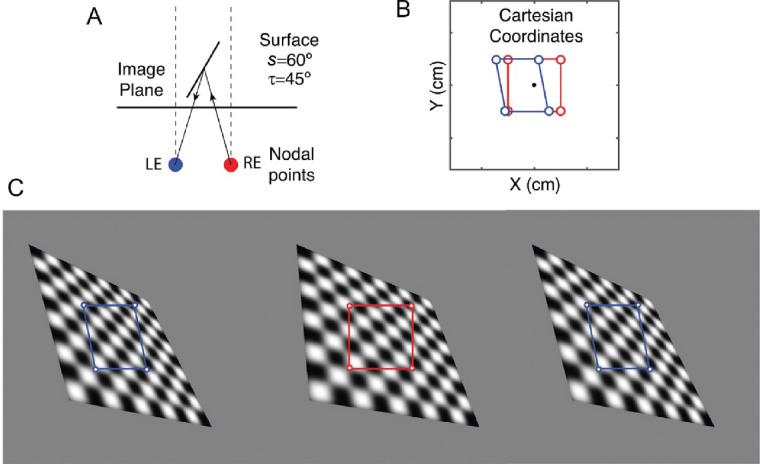
Image differences in Cartesian coordinates. (**A**). Viewing geometry. (**B**) A square image patch in the right eye view (red) and the back projection of its boundary to the left eye (blue), in Cartesian coordinates centered on the cyclopean eye. For any planar surface, a square region in one eye projects to a parallelogram in the other eye. (**C**) Stereo pair of surfaces and patches in each eye. Fuse images to see a surface with a slant of 60 degrees and a tilt of 45 degrees (uncrossed pair: left and center, crossed pair: center and right).

The LPM observer is consistent with the hypothesis that early binocular receptive fields are explicitly coding both the location and pattern disparities that are produced by the backward and forward projection of planar surfaces. For example, Figure 13 shows the binocular receptive fields that would respond best to a sinewave textured surface at 100 cm, with a slant of 60 degrees, for five different tilts. To emphasize the shape differences between left and right receptive fields, the imaging plane was set to the same distance as the surface (100 cm). If the imaging plane was instead set at plus or minus 17 mm (the location of the retinas), then the left and right receptive fields would also differ in location. When the surface tilt is 0 degrees (see Figure 1B), the left and right receptive fields differ primarily in preferred frequency. When the surface tilt is 90 degrees the left and right receptive fields differ primarily in preferred orientation. For other tilts, there are frequency, orientation, and shear differences (see also Figure A3). The third column shows the differences between the left and right receptive fields. Figure 13B shows how the total energy of the difference between the left and right receptive fields varies with slant and tilt. The energy tends to increase with slant and decrease with tilt, although this latter effect is quite small for low slants.

Although the LPM and the PM observers demonstrate that pattern disparities provide substantial additional information for slant discrimination and estimation (see also, Jones & Malik, 1992; Devernay & Faugeras, 1994; Super & Klarquist, 1997; Li & Zucker, 2005; Ogale & Aloimonos, 2005; Vidal-Naquet & Gepshtein, 2012), there remains uncertainty about the extent to which pattern disparities are explicitly encoded in the early visual system.

Single unit recordings in the primary visual cortex of monkeys and cats have found populations of neurons with binocular receptive fields that are consistent with pattern disparity in orientation (Bridge & Cumming, 2001) and spatial frequency (Sanada & Ohzawa, 2006). However, modeling with generalized versions of the disparity energy model originally introduced by Ohzawa et al. (1990) showed that most of the useful disparity information is captured by standard location-disparity detectors (Bridge, Cumming, & Parker, 2001; Nienborg, Bridge, Parker, & Cumming, 2004). Nonetheless, these models do not consider all of the pattern disparities associated with planar surfaces (see Figure 13) and do not quantify how much the reliability of location-disparity estimates increases when pattern disparities are estimated simultaneously (see Figure 4). In addition, the possible benefits of including pattern-disparity information may better emerge in models of population decoding that pool efficiently over all the relevant neurons and hence large spatial areas (Bridge & Cumming, 2008; Greenwald & Knill, 2009; Kato, Baba, Sasaki, & Ohzawa, 2016).

To be more concrete, consider a binocular neuron having any common receptive field shape and location in one of the eyes (e.g. a vertical Gabor receptive field). For any surface orientation, distance, and eye pose, the corresponding receptive field in the other eye can be determined by back-projecting that the receptive field to the surface and then forward projecting to the other eye. Most of the differences between the receptive fields in the two eyes will be modest and described to good approximation by differences in location, spatial frequency, and orientation (e.g. see Figure 13). Hence, the variation in left and right eye receptive fields measured in V1 is likely to cover a substantial fraction of the range in variation produced by typical variations in surface orientation, distance, and eye pose (keep in mind that there are thousands of V1 neurons covering each image location). If cortical circuits combine these V1 responses efficiently (perhaps together with efferent-copy signals), they would be using the information captured by the LPM observer. On the other hand, if cortical circuits simply average (pool) the responses over all the V1 neurons at a given location having a given preferred horizontal disparity, then they would be using the information captured by the LFM observer. This information would be substantially poorer for V1 neurons having membrane-potential receptive fields (receptive fields prior to the spiking nonlinearity) larger than 0.5 degrees (see Figure 8), which is true for some V1 neurons in the fovea, many neurons in the parafovea, and probably most neurons in the near periphery (Tsao, Freiwald, Knutsen, Mandeville, & Tootell, 2003; Nienborg et al., 2004; Palmer et al., 2012; Chen, Ko, Zemelman, Seidemann, & Nauhaus, 2020).

Psychophysical studies have also not yet provided a clear picture of the role of pattern disparities. Studies that have focused on pattern disparities have revealed that it is difficult to reject simple LFM type models (Halpern et al., 1996; Hibbard & Langley, 1998; Heeley, Scott-Brown, Reid, & Maitland, 2003; Greenwald & Knill, 2009). Studies that have either reported evidence for LFM-type computations (Banks et al., 2004; Filippini & Banks, 2009; Allenmark & Read, 2011; Goutcher & Hibbard, 2014) or for LPM-type computations (Hibbard et al., 2002), generally have not directly compared the models. Thus, although the LFM-type models are more parsimonious because of their simplicity, there does not appear to be compelling evidence either for or against the visual system's use of pattern disparities.

Finally, it is important to note that whereas the PM model is approximately optimal for estimating the slant and distance of planar textured surfaces (with uncorrelated image noise), it is not optimal under real-world conditions, where many surfaces (except the ground plane) are non-planar and where there are half occlusions (points with no corresponding point in the other eye). More sophisticated computations are required in natural 3D scenes (Scharstein & Szeliski, 2002; Hirschmuller & Scharstein, 2007). The human visual system is likely to be much more sophisticated than the models considered here. It may be possible to generalize the Bayesian framework described in the Methods to handle non-planar surfaces and half occlusions.

There are additional research approaches that could be useful for discriminating between the different models described here. One approach is to vary stimulus parameters, such as the size and spatial-frequency content of the test patches and the range of test slants and tilts, to find those parameter values where the models make the biggest differences in the predicted pattern of slant thresholds. Testing with these parameter values should better differentiate between the models. Another approach is to consider what specific computations are optimal for natural images. For example, Burge and Geisler (2014) used accuracy maximization analysis (AMA) of natural images to determine the set of vertically oriented binocular receptive fields that are optimal as a population for estimation of local disparity (Geisler, Najemnik, & Ing, 2009; see also Burge & Jaini, 2017, and Jaini & Burge, 2017). The receptive fields in this population share many properties with receptive fields measured in the visual cortex. It should be possible to perform a similar analysis to determine the optimal population of binocular receptive fields for estimating surface slant or surface slant and distance of natural images. This type of normative analysis could be used to guide investigations of receptive field properties in the cortex that may capitalize upon the pattern disparities that result from binocularly viewed slanted surfaces.

### Coordinate systems and the representation of surface orientation

Throughout this paper, estimates of surface orientation and distance were made in a head-centric coordinate system, with gaze direction parallel to the cyclopean axis. Keeping the same eye pose, the matching computations could also be used to make estimates in direction-centered coordinate systems. For example, the direction-centered coordinate system for each image location could be defined as one aligned with the axis passing through that image location and the origin. Direction-centered coordinate systems are illustrated in Figure 3C. A plausible hypothesis is that local surface orientation and distance are initially estimated in local direction-centric coordinates. These estimates might be used when the observer is tasked with reporting local surface slants with respect to a direction (without changing gaze). These local measurements might then be mapped into head-centric coordinates, where they are grouped into 3D surfaces. Importantly, the grouping rules are often simpler in head-centric coordinates than in direction-centric coordinates. For example, with the representation in Figure 3B, one can use simple similarity grouping to group estimated local slants into a 3D plane, but similarity grouping would not work for the representation in Figure 3C. In addition, as Backus et al. (1999) note, mapping into head-centric and body-centric representations are important for implementing motor behaviors.

## Conclusion

Stereo slant discrimination performance was measured for accurately rendered textured surfaces designed so that performance is dominated by binocular-disparity cues. We compared human performance with model observers (PM and LPM) that simultaneously estimate distance and surface orientation without directly estimating disparities and with a model observer (LFM) that first directly estimates disparities and then combines those to estimate distance and surface orientation. We found that the pattern of human slant-discrimination thresholds was predicted equally well by all three models. However, even though the LFM model is the simplest and most parsimonious, we find that the LPM models perform substantially better at slant discrimination; hence, there may have been evolutionary pressure to incorporate similar computations into the early visual system. We also mention additional research approaches that may better differentiate between the models.

## Figures and Tables

**Figure A2.**
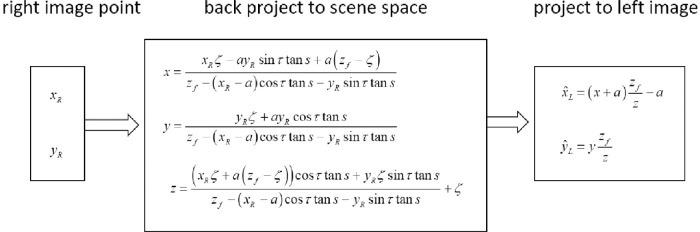
Equations for projecting a point in the right image to a point in the left image given a planar surface at an intercept distance of ζ having a slant of *s* and a tilt of τ. The distance of the image plane to the nodal point of each eye/camera is *z_f_* and the separation between the nodal points is 2*a*. The predicted image value (e.g. gray level) at left image location ${\hat{x}}_{L},{\hat{y}}_{L}$ is the image value at *x_R_*,*y_R_*.

## Appendix

### Projection of planar surfaces

Here, we derive the exact equations for mapping a point in the right image to the corresponding point in the left image given a planar surface at an intercept distance of ζ, with a slant of *s* and a tilt of τ, for the imaging geometry illustrated in Figure 3. The equations are shown in Figure A2. These equations are for arbitrary slant, tilt, and distance, but for the current experiments the tilt was set to zero.

The planar matching (PM) is implemented by applying these equations to all the points in an image patch in the right eye for each possible intercept distance, slant, and tilt of the planar surface. In other words, the predicted image value (e.g. gray level) at the predicted left image location $\left({\hat{x}}_{L},{\hat{y}}_{L}\right)$ is the image value at (*x_R_*,*y_R_*): ${\hat{I}}_{L}\left({\hat{x}}_{L},{\hat{y}}_{L}|s,\zeta \right)=I_{R}\left(x_{R},y_{R}\right)$. The estimate of distance and surface orientation are the values of distance, slant, and tilt that give the most accurate prediction of the left-eye image (smallest mean squared error).

For the PM model the image patch is the whole right image of the test plane. For local planar matching (LPM) and local frontoparallel matching (LFM), the right image patch is a smaller fixed-size image patch. In addition, for the LPM and LFM models, the equations are expressed in terms of distance *z* rather than intercept distance ζ(see Figure 5). The equations in Figure A2 can be expressed in terms of distance by setting ζ = *z* − *x* tan *s* cos τ + *y* tan *s* sin τ. Note that in the present experiment, where tilt is zero, ζ = *z* − *x* tan *s*, and that for the LFM model ζ = *z*, because of the assumption that *s* = 0.

The equations in Figure A2 are standard projective geometry, but are derived here to provide compact equations that are easy to apply. To derive the equations in Figure A2, we first note that if the nodal point is at the origin in 3D Euclidean space (as in Figure 3), then the standard equations for perspective projection are

(A1) $$\begin{array}{c} x^{'}=x\frac{z_{f}}{z}\; \end{array}$$

(A2) $$\begin{array}{c} y^{'}=y\frac{z_{f}}{z}\; \end{array}$$

where *z_f_* is the distance from the nodal point to the image plane, and (*x*′, *y*′) is the point in the image plane. If the nodal point is shifted to the left by a distance of *a* (left eye point in Figure 3), then the point in the image plane (*x_L_*,*y_L_*) is given by

(A3) $$\begin{array}{c} x_{L}=\left(x+a\right)\frac{z_{f}}{z}-a\; \end{array}$$

(A4) $$\begin{array}{c} y_{L}=y\frac{z_{f}}{z}\; \end{array}$$

Equations A3 and A4 are the equations in the right panel of Figure A2. If the nodal point is shifted to the right, then the point in the image plane is given by

(A5) $$\begin{array}{c} x_{R}=\left(x-a\right)\frac{z_{f}}{z}+a\; \end{array}$$

(A6) $$\begin{array}{c} y_{R}=y\frac{z_{f}}{z}\; \end{array}$$

The slant, tilt, and distance at a surface point (*x*, *y*, *z*) are defined here in global Euclidean coordinates as the slant, tilt, and intercept distance (*s*, τ, ζ) of the plane passing through that surface point (Figure 3), where the slant, tilt, and distance are with respect to the cyclopean axis. The intercept distance ζ is the intercept of the plane with the cyclopean axis. The slant *s* is defined as the magnitude of the angle (0–90 degrees) between the surface normal and the cyclopean (or optic) axis, and the tilt τ is defined as the direction (−180 degrees to –180 degrees) around that axis in which distance is changing most rapidly (the counter-clockwise angle of the parallel projection of the surface normal vector; see Figure 1). Using these definitions, the equation of the plane is

(A7) $$\begin{array}{c} z=x\cos\tau \tan s+y\sin\tau \tan s+\zeta \; \end{array}$$

Specifically, the textbook definition of the equation of a plane is **n** · (**x** − **x**_*p*_) = 0, where **n** is the normal vector at location**x**_ζ_ = (0, 0, ζ). The normal vector is obtained by rotating the unit normal vector of the frontoparallel plane, (0, 0, −1), around the vertical (*y*) axis by angle *s*, and then rotating the resulting vector around the distance (*z*) axis by angle τ. Substituting the rotated normal vector into the equation for a plane gives Equation A7.

To derive the equations for back projection, we first rearrange Equations A5 and A6:

(A8) $$\begin{array}{c} x=\frac{z}{z_{f}}\left(x_{R}-a\right)+a\; \end{array}$$

(A9) $$\begin{array}{c} y=\frac{z}{z_{f}}y_{R}\; \end{array}$$

Substituting Equation A7 into Equation A8 we have

(A10) $$x=\frac{\cos\tau \tan sx+\sin\tau \tan sy+\zeta }{z_{f}}\left(x_{R}-a\right)+a$$

Subtracting *a* from both sides of Equation A8 and then taking the ratio with Equation A9 we get

(A11) $$y=\left(x-a\right)\frac{y_{R}}{\left(x_{R}-a\right)}$$

Substituting Equation A11 into Equation A10 gives

(A12) $$\;\;\;\;\;x=\frac{\zeta +x\cos\tau \tan s+\left(x-a\right)\frac{y_{R}}{\left(x_{R}-a\right)}\sin\tau \tan s}{z_{f}}\left(x_{R}-a\right)+a$$

Simplifying this equation gives the expression for *x* in Figure A2,

(A13) $$x=\frac{\left(x_{R}-a\right)\zeta -ay_{R}\sin\tau \tan s+az_{f}}{z_{f}-\left(x_{R}-a\right)\cos\tau \tan s-y_{R}\sin\tau \tan s}$$

Next substitute Equation A7 into Equation A9

(A14) $$y=\frac{\cos\tau \tan sx+\sin\tau \tan sy+\zeta }{z_{f}}y_{R}$$

Rearranging Equation A11 gives

(A15) $$x=y\frac{x_{R}-a}{y_{R}}+a$$

Substituting Equation A15 into Equation A14 and simplifying gives the expression for *y* in Figure A2:

(A16) $$y=\frac{y_{R}\zeta +y_{R}a\cos\tau \tan s}{z_{f}-\left(x_{R}-a\right)\cos\tau \tan s-y_{R}\sin\tau \tan s}$$

Finally, substituting Equations A13 and A16 into Equation A7 and simplifying gives

(A17) $$\;\;\;\;\;\;\;\;\;z=\frac{\left(x_{R}\zeta +\left(z_{f}-\zeta \right)a\right)\cos\tau \tan s+y_{R}z_{p}\sin\tau \tan s}{z_{f}-\left(x_{R}-a\right)\cos\tau \tan s-y_{R}\sin\tau \tan s}+\zeta$$

Equations A13, A16, and A17 are the back-projection equations in Figure A2.

Figure A3 illustrates the backward and forward projections for a surface with a slant of 60 degrees and a tilt of 45 degrees. Note that a square region in one image always projects to a parallelogram in the other image.
